# Intermedin protects against sepsis by concurrently re-establishing the endothelial barrier and alleviating inflammatory responses

**DOI:** 10.1038/s41467-018-05062-2

**Published:** 2018-07-06

**Authors:** Fei Xiao, Denian Wang, Lingmiao Kong, Min Li, Zhongxue Feng, Bingxing Shuai, Lijun Wang, Yong’gang Wei, Hongyu Li, Sisi Wu, Chun Tan, Huan Zhao, Xuejiao Hu, Jin Liu, Yan Kang, Xuelian Liao, Yan Zhou, Wei Zhang

**Affiliations:** 1 Molecular Medicine Research Center, State Key Laboratory of Biotherapy/Collaborative Innovation Center for Biotherapy, West China Hospital, Sichuan University, Chengdu, 610041 China; 2 Department of Intensive Care Unit of Gynecology and Obstetrics, West China Second University Hospital, Sichuan University, Chengdu, 610041 China; 3 Department of Intensive Care Unit, West China Hospital, Sichuan University, Chengdu, 610041 China; 4 Department of Liver Surgery, West China Hospital, Sichuan University, Chengdu, 610041 China; 5 Department of Laboratory Medicine, West China Hospital, Sichuan University, Chengdu, 610041 China; 6 Department of Anesthesiology, West China Hospital, Sichuan University, Chengdu, 610041 China

## Abstract

Sepsis is a life-threatening condition caused by dysregulated host responses to infection. Widespread vascular hyperpermeability and a “cytokine storm” are two pathophysiological hallmarks of sepsis. Here, we show that intermedin (IMD), a member of the calcitonin family, alleviates organ injury and decreases mortality in septic mice by concurrently alleviating vascular leakage and inflammatory responses. IMD promotes the relocation of vascular endothelial cadherin through a Rab11-dependent pathway to dynamically repair the disrupted endothelial junction. Additionally, IMD decreases inflammatory responses by reducing macrophage infiltration via downregulating CCR2 expression. IMD peptide administration ameliorates organ injuries and significantly improves the survival of septic mice, and the experimental results correlate with the clinical data. Patients with high IMD levels exhibit a lower risk of shock, lower severity scores, and greatly improved survival outcomes than those with low IMD levels. Based on our data, IMD may be an important self-protective factor in response to sepsis.

## Abstract (web summary)

Sepsis is a life-threatening condition. Here, the authors show that intermedin alleviates organ injury and decreases mortality in septic mice by concurrently alleviating vascular leakage and inflammatory responses. Patients with high intermedin levels exhibit a low risk of shock, lower severity scores, and greatly improved survival outcomes.

## Introduction

Sepsis is a clinical syndrome occurring in patients following infection or injury, which is linked to multi-organ dysfunction and inadequate tissue perfusion in many cases, and is the major cause of death in intensive care units, with an associated mortality rate ranging from 30 to 70%^1–3^. Research on the pathogenesis of sepsis has traditionally focused on inflammatory responses. However, the repeated failure of anti-inflammatory agents suggests that some fundamental knowledge is lacking in our current understanding of sepsis. According to recent studies, vascular endothelial hyperpermeability is not merely the by-product of sepsis but rather a major contributor to its morbidity and mortality^4–6^. Oedema typically develops in patients with sepsis, indicating widespread vascular leakage throughout the body. The accumulation of fluid in the interstitium and parenchyma increases the distance required for the diffusion of oxygen and compromises micro-vessel perfusion because of increased interstitial pressure, resulting in multi-organ dysfunction^5^. Because all blood vessels are lined with endothelial cells (ECs), the leakage and oedema suggest endothelial barrier dysfunction^5^. Vascular endothelial cadherin (VE-cadherin, VEC) is the major component of adherens junctions (AJs) at cell–cell contacts and controls endothelial monolayer permeability^7,8^. The displacement of VEC from cell–cell contacts induces gaps between ECs, leading to increased permeability. Thus, strategies designed to repair the VEC complex at endothelial cell–cell contacts may resolve the vascular leakage and provide patients with sepsis the opportunity to recover from organ dysfunction caused by oedema and inadequate tissue perfusion.

Intermedin (IMD), a member of the calcitonin family^9,10^, plays a role in maintaining endothelial barrier function^11–14^. IMD abolishes the increase in pressure-induced endothelial permeability in an isolated lung model^11^, antagonizes the thrombin-induced permeability of a human umbilical vascular endothelial cell (HUVEC) monolayer by stabilizing the VEC complex^12^, and stabilizes the endothelial barrier function in vitro and attenuates ventilator-induced lung injury in mice^13^. Thus, IMD may have a protective role in sepsis by decreasing endothelial hyperpermeability. IMD also exerts anti-inflammatory effects on an insulin-resistant model and an IgA nephropathy model^15,16^, indicating that IMD may also affect the inflammatory response in patients with sepsis.

In the present study, we aim to explore whether IMD expression is altered during sepsis, whether IMD alleviates the widespread vascular leakage and protects against the cytokine storm and the inflammatory mediators-induced endothelial barrier dysfunction, and whether the administration of IMD peptide is beneficial for the survival of septic mice. We show that IMD protects against sepsis by concurrently re-establishing the endothelial barrier and alleviating inflammatory responses. Our study may provide novel insights that improve our understanding of the mechanism of endothelial barrier stabilization and inflammatory response regulation during sepsis.

## Results

### IMD expression was markedly increased in septic mice

**Fig. 1:**
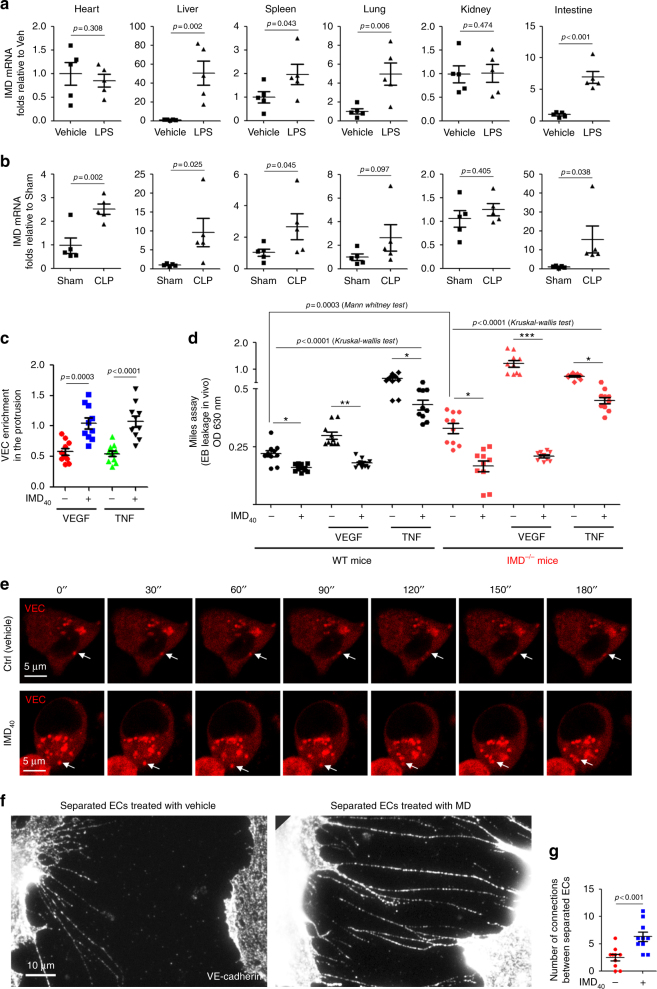
IMD was significantly up-regulated in septic mice, repairing the endothelial junction by promoting VEC relocation. **a, b** The Balb/c mice received intraperitoneally (i.p.) injection of LPS (24 mg/kg) or vehicle (saline), or had sham or CLP surgery. After 9 h, mice were sacrificed, and level of IMD mRNA in major organs was measured by real-time RT-PCR. **c** The HUVEC monolayer was treated with vehicle (PBS), IMD_40_ (2 μM), VEGF (50 ng/ml), TNF-α (20 ng/ml) alone, or treated with VEGF or TNF-α for 2 h followed by treatment of IMD_40_. The density of VEC signal referred to F-actin at the cell–cell contact was quantified using 10 randomly chosen fields from two experiments. **d** The Miles assay was performed as described in Methods. The Evans-Blue (EB) leakage (OD 630 nm) of WT or IMD^−/−^ mice was quantified (*n* = 10 mice). **e** HUVECs were transfected with the lentivirus that expresses mCherry-tagged VEC (red), and a time-lapse photography was performed with a 30-s interval. The arrows indicate the location of a single VEC endosome at each time point. **f** The completely separated ECs were re-connected by the extended filopodia with the presence of IMD_40_. **g** The number of anastomosed filopodia connecting two completely separated HUVECs was quantified using 10 randomly chosen fields from two experiments. The data of (**a–d, g**) were presented as scatter plots with mean ± SEM. Significance was assessed by Mann–Whitney test (**a–c,g**) and one-way ANOVA (Kruskal–Wallis test) followed by non-parametric Dunn’s post-hoc analysis (**d**)

In the present study, we used two classic septic mouse models, lipopolysaccharide (LPS)-induced endotoxaemia and a caecal ligation and puncture (CLP)-induced septic model, to investigate the role of IMD in sepsis. The major organs were collected 9 h after LPS injection or CLP surgery and subjected to real-time PCR analysis. Compared with the vehicle-treated mice, the LPS-injected mice displayed increased mean levels of the IMD mRNA in liver, spleen, lung, and intestine, 50.8- (*p* = 0.002), 1.9- (*p* = 0.043), 5- (*p* = 0.006), and 6.9-fold (*p* < 0.001), respectively (Fig. 1a, Mann–Whitney test). Levels of the IMD mRNA in the heart, liver, spleen, and intestine were increased 2.5- (*p* = 0.002), 9.7- (*p* = 0.025), 2.6- (*p* = 0.045), and 15.6-fold (*p* = 0.038), respectively in CLIP mice compared with control group mice (Fig. 1b, Mann–Whitney test). The immunohistochemistry and Western blot results showed similar trends in IMD protein expression in the LPS and CLP mice (Supplementary Fig. 1, 2).

### IMD dynamically repaired the AJ gaps

The significant upregulation of IMD suggested that IMD might be involved in the pathogenesis of sepsis. In subjects with sepsis, inflammatory mediators, such as tumour necrosis factor (TNF)-α^17–19^ and vascular endothelial growth factor (VEGF)^20–22^, are known to cause endothelial barrier dysfunction by inducing VEC complex dissociation. Both VEGF and TNF-α induced AJ gaps at cell–cell contacts (Supplementary Figs. 3a). Treatment with a mature form of the IMD peptide (IMD_40_, a 40-amino acid peptide from residues 8 to 47) alone did not affect the pre-established AJs but repaired both the VEGF- and TNF-α-induced AJ gaps by promoting the regeneration of the zipper-like VEC complex (Supplementary Figs. 3a). Double staining of HUVECs with an anti-VEC antibody and phalloidin (Supplementary Figs. 3a, lower panels) revealed that IMD_40_ promoted the enrichment of VEC in the protrusions between adjacent cells (Fig. 1c), which facilitated the formation of new AJs by the cells. Based on the results of the Miles assay, IMD_40_ not only alleviated the VEGF- and TNF-induced vascular leakage but also decreased the basal leakage in vivo, indicating that the IMD-reconstructed AJs were functional (Fig. 1d).

We employed the CRISPR/Cas9 system to generate IMD knockout (IMD^−/−^) mice, which were ostensibly viable and fertile. However, the Miles assay showed a marked increase in the basal EB leakage in IMD^−/−^ mice, and VEGF induced more severe leakage in the IMD^−/−^ mice than in the WT mice. Systematic IMD_40_ administration markedly reduced the vascular leakage in both IMD^−/−^ and WT mice (Fig. 1d). Thus, although the IMD^−/−^ mice appeared healthy, they may have exhibited some pathogenic changes in the endothelial barrier that led to vascular hyperpermeability.

As shown in our previous report^14^, IMD increases VEC expression in ECs. Here, we sought to explore whether IMD induces VEC endosome movement in ECs. We transfected HUVECs with a lentivirus expressing mCherry-tagged VEC and found that IMD_40_ not only increased the amount of VEC endosomes but also promoted their re-distribution in the cytoplasm (Fig. 1e). In addition, IMD_40_ significantly increased the enrichment in VEC endosomes in the free filopodia on the cell surface, whereas the VEGF treatment reduced VEC enrichment (Supplementary Fig. 3b,c). Furthermore, IMD_40_ promoted the formation of connections between the completely separated ECs through the extended filopodia in which the VEC endosomes were enriched, strengthening the connection (Fig. 1f,g). IMD_inh_, a truncated form of IMD_40_ (from residues 17 to 47) that was used as a competitive inhibitor of IMD^23^, exerted opposite effects (Supplementary Fig. 4a–c). Based on these results, IMD promoted the re-establishment of new AJs between detached ECs by promoting the relocation of VEC endosomes to the filopodia and protrusions at cell–cell contacts and did not simply prevent the established AJs from dissociating.

### Rab11 facilitated the IMD-induced VEC re-localization

**Fig. 2:**
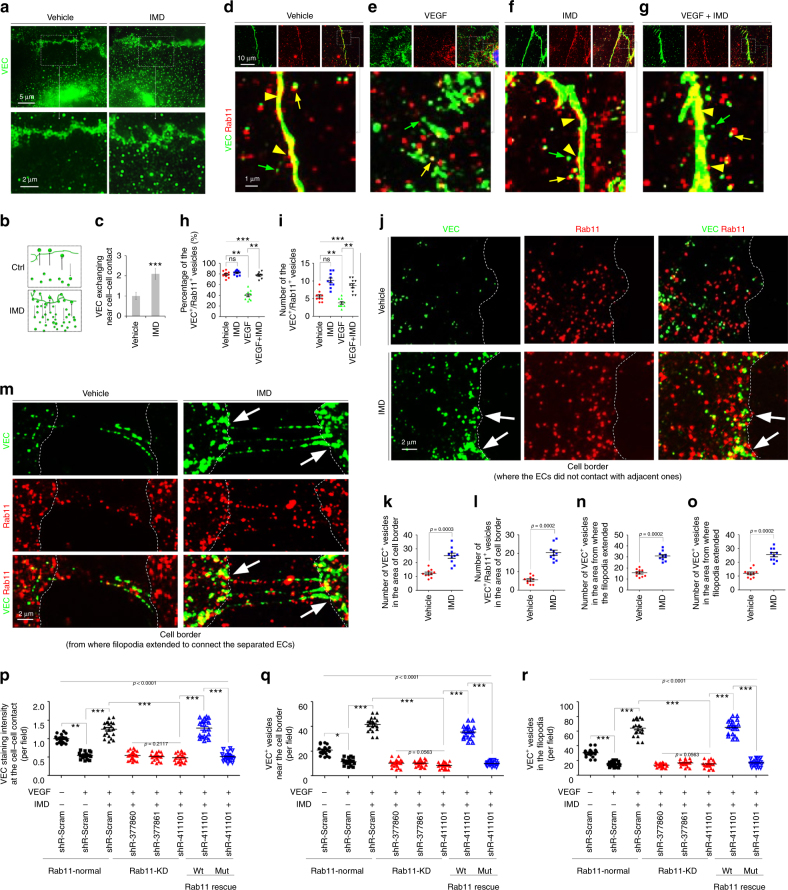
Rab11 facilitates the IMD-induced VEC re-localization. **a** The HUVECs treated with vehicle (PBS) or IMD_40_ (2 μM) were stained with VEC (green). **b** The schematic of the VEC endosome exchange at the cell–cell contact. **c** The VEC exchange was quantified using 10 randomly chosen fields from two experiments and expressed relative to the vehicle group. **d–g** The Antibody feeding assay was performed as described in Methods. The HUVEC monolayer was treated with PBS, IMD, VEGF, or VEGF plus IMD. The internalized VEC endosomes were detected by pre-incubation of anti-VEC (green), and double-stained with anti-Rab11 (red). The yellow arrows indicate the VEC^+^/Rab11^+^ double-positive vesicles, the yellow arrowheads indicate the Rab11^+^ vesicles fused to the VEC-complexes, and the green arrows indicate the VEC^+^ endosomes that did not co-localized with Rab11. **h, i** The percentage of VEC^+^/Rab11^+^ vesicles in total VEC^+^ vesicles and the absolute number of them were quantified using 10 randomly chosen fields. **j, m** The Antibody feeding assay showed the VEC^+^ and Rab11^+^ vesicles at the cell border of HUVECs. **k, l, n, o** The number of VEC^+^ or VEC^+^/Rab11^+^ double-positive vesicles in the area of cell border where cells did not contact with adjacent ones (**k,l**), or the area from where the filopodia extended to connect the separated ECs (**n, o**) were quantified using 10 randomly chosen fields. **p, q, r** The HUVECs were transfected with the shRNAs of Rab11 (shR-377860, shR-377861, shR-411101), followed by the treatment of VEGF with or without IMD_40_. shR-411101 that targets the 3′-UTR of Rab11 was rescued by transfecting the vector that expresses wild type or mutant Rab11 lacking 3′-UTR. The VEC staining intensity at the cell–cell contact, and the VEC^+^ vesicles at the cell border and in the filopodia were quantified using 10 randomly chosen field. All data were presented as scatter plots with mean ± SEM. Significance was assessed by Mann–Whitney test (**c, k, l, n, o**) and Kruskal–Wallis test followed by non-parametric Dunn’s post-hoc analysis (**p–r**)

We further investigated the mechanism by which IMD promoted VEC re-localization. As shown in Fig. 2a, the number of VEC endosomes located near the region of the cell–cell contact was significantly increased after treatment with IMD, indicating an increase in the exchange/recycling between the free VEC endosomes and the VEC complex at the membrane (Fig. 2b, c). E-cadherin, the key component of epithelial cell junctions (VEC is the corresponding molecule in EC junctions), is recycled to the plasma membrane with the help of Rab11, which is a key mediator that facilitates the canonical recycling of membrane proteins^24,25^. We then asked whether Rab11 also mediated the IMD-induced transport of VEC vesicles. Fluorescent microscopy detected abundant Rab11^+^ vesicles in the cytoplasm of ECs (Supplementary Fig. 5). Interestingly, based on the results of the antibody feeding assay, most of the VEC^+^ vesicles located near cell–cell contacts co-localized with Rab11^+^ vesicles (Fig. 2d, h; yellow arrow), and a substantial number of Rab11^+^ vesicles co-localized with the VEC complexes (Fig. 2d; yellow arrowhead). Thus, Rab11 may be a major factor contributing to promote steady-state VEC endosome recycling to the membrane.

VEGF promotes the rapid endocytosis of VEC, thereby disrupting the VEC complex^8,26^. The antibody feeding assay detected the VEGF-induced AJ gaps and the increased number of VEC endosomes located near cell–cell contacts. Only a small part of the internalized VEC endosomes co-localized with the Rab11^+^ vesicles (Fig. 2e, h), and the number of VEC^+^/Rab11^+^ double-positive vesicles was significantly decreased (Fig. 2e, i), indicating that most of the internalized VEC vesicles observed following treatment with VEGF were not recycled to the membrane to rebuild the VEC complex. On the other hand, IMD significantly induced the recruitment of Rab11^+^ vesicles to the internalized VEC^+^ endosomes and to the newly formed zipper-like VEC complex (Fig. 2f, g), increasing both of the ratio and the number of VEC^+^/Rab11^+^ double-positive endosomes (Fig. 2h, i). In addition, after treatment with IMD, the Rab11^+^/VEC^+^ endosomes preferentially accumulated at cell borders lacking EC contacts (Fig. 2j–l), and cell borders from which filopodia extended to connect the separated ECs (Fig. 2m, o). Based on these results, the Rab11^+^ vesicles may facilitate the transport of VEC endosomes to the cell border and filopodia to strengthen the connections between the adjacent but separate ECs, and IMD may use this mechanism to repair the AJ gaps.

We performed an RNA interference experiment to test this hypothesis (Supplementary Fig. 6 and 7a–c). Rab11 silencing significantly impeded the ability of IMD to repair the VEGF-induced AJ gaps (Supplementary Fig. 7 and Fig. 2p). In addition, the recruitment of VEC endosomes to the cell border (Fig. 2q) and the filopodia (Fig. 2r) was markedly decreased, suggesting that the IMD-induced VEC endosome recycling depended on Rab11. We used an shRNA that targets the 3′-UTR to silence Rab11 and rescued Rab11 expression by transfecting the vector that expresses wild type Rab11 lacking the 3′-UTR to exclude possible off-target effects (Supplementary Fig. 7, right panels). IMD re-acquired the ability to repair the AJ gaps and to relocate VEC in cells transfected with wild type Rab11 (Fig. 2p–r). However, no rescue effect was observed when cells were transfected with a dominant negative form of Rab11 (Rab11S25N), indicating that the inactive form of Rab11 did not facilitate the IMD-induced VEC transport (Fig. 2p–r).

### IMD decreased vascular leakage in septic mice

**Fig. 3:**
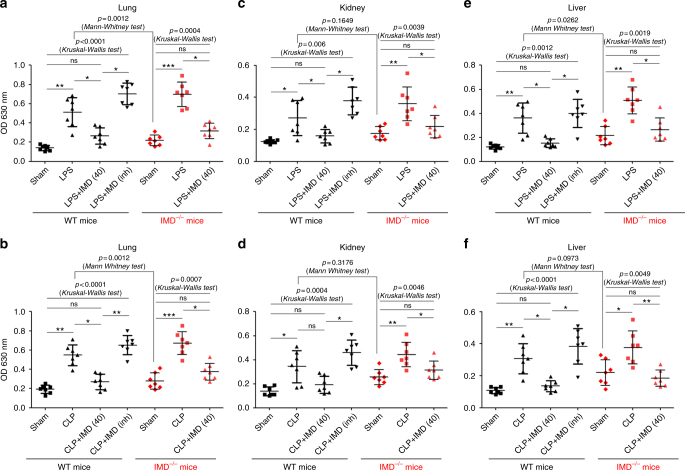
IMD alleviated the vessel leakage in lung, kidney, and liver. C57/BL6 female (8–10 weeks, 20–25 g, housed in SPF conditions, *n* = 7) WT or IMD^−/−^ mice were injected IMD_40_ (0.5 mg/kg) or IMD_inh_ (1 mg/kg) subcutaneously 1 h before LPS administration (24 mg/kg) or CLP surgery. The EB leakage of lung, kidney, and liver from LPS-treated mice (**a–c**) or CLP-treated mice (**d–f**) was determined by optical density (OD 630 nm). Data was presented as scatter plots with mean ± SD (*n* = 7 per group). Significance was assessed by Kruskal–Wallis test followed by non-parametric Dunn’s post-hoc analysis. The control mice (WT or IMD^−/−^) was compared by Mann–Whitney test separately

The breakdown of pulmonary and renal vascular barriers and fluid leakage are vitally involved in the subsequent development of multiple organ dysfunction in subjects with sepsis. Here, we tested whether IMD had a role in preventing hyperpermeability in vivo. The Evans Blue (EB) leakage in the lung was significantly increased 5 h after LPS administration or CLP surgery. The administration of IMD_40_ markedly decreased the vascular permeability in both the LPS and CLP mice but did not decrease this parameter below the baseline level. In contrast, IMD_inh_ exacerbated the vessel leakage in both the LPS and CLP models (Fig. 3a, b). The bronchus bronchoalveolar lavage assay yielded similar results (Supplementary Fig. 8). In addition, in the kidney and the liver, IMD exerted a similar effect on alleviating vascular hyperpermeability (Fig. 3c–f). IMD depletion resulted in spontaneous vessel leakage, and exacerbated the LPS- or CLP-induced leakage compared with the WT mice (Fig. 3). These results were consistent with observations that IMD successfully re-established the endothelial barrier.

### IMD ameliorated the inflammatory response of septic mice

**Fig. 4:**
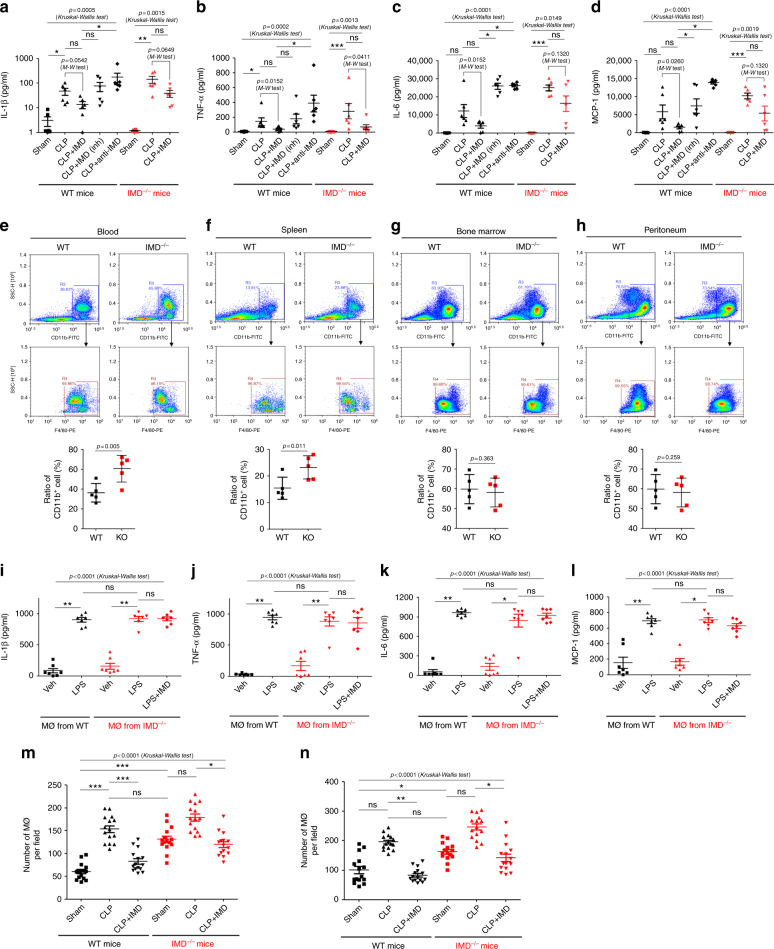
IMD ameliorated the inflammatory response. **a–d** IMD_40_ (0.5 mg/kg), or IMD_inh_ (1 mg/kg), or anti-IMD mAb (2.5 mg/kg) was injected into WT or IMD^−/−^ mice 1 h before CLP or sham surgery. The blood samples were harvested 9 h after surgery. The concentration of IL-1β (**a**), IL-6 (**b**), TNF-α (**c**), and MCP (**d**) was determined by ELISA. **e–h** The macrophage profile on steady state of blood, spleen, bone marrow, and peritoneum. Gating strategy: using CD11b and F4/80 to identify the macrophage. The lowest panels: the macrophage profile (%) was presented as scatter plots with mean ± SEM (*n* = 5 mice). **i–l** The peritoneal macrophages were isolated from WT or IMD^−/−^ mice, and tested for cytokine productions after the stimulation of LPS. **m, n** The macrophage infiltration in liver and lung 9 h after surgery was quantified. Number of macrophages per field was presented as scatter plots with mean ± SEM using 15 randomly chosen fields from three mice. Significance was assessed by Mann–Whitney test **(e-h)** or Kruskal–Wallis test followed by non-parametric Dunn’s post-hoc analysis **(a–d, m, n)**

TNF-α, interleukin-1β (IL-1β), IL-6, and monocyte chemotactic protein-1 (MCP-1) are hallmark inflammatory mediators of sepsis that constitute the cytokine storm. CLP surgery greatly increased the serum levels of TNF-α, IL-1β, IL-6, and MCP-1 in WT mice (Fig. 4a–d). Interestingly, IMD_40_ administration significantly decreased CLP-induced cytokine production in both WT and IMD^−/−^ mice, whereas IMD inhibition or IMD depletion further exacerbated the cytokine storm (Fig. 4a–d). Based on these results, IMD alleviates the inflammatory response in sepsis. Macrophages and neutrophils are the two main sources of the cytokine storm. IMD depletion increased the macrophage ratio in the blood and spleen (Fig. 4e–h), although the neutrophil profiles were not affected (Supplementary Fig. 9). Neither IMD depletion nor the IMD_40_ treatment affected cytokine production in isolated macrophages (Fig. 4i–l). Thus, the IMD-induced systemic decrease in cytokine production is likely due to the reduced number of macrophages and not the inhibition of cytokine production by macrophages. In addition, immunohistochemical staining revealed a significantly greater number of infiltrating macrophages in the liver and lungs of IMD^−/−^ mice but not WT mice that underwent CLP surgery, whereas IMD_40_ administration reduced macrophage infiltration in both WT and IMD^−/−^ mice (Supplementary Fig. 10 and Fig. 4m, n). IMD depletion affected neither the phagocytic capacity of macrophages nor the microbial clearance in the circulation (Supplementary Fig. 11), indicating that although IMD ameliorated the inflammatory response, it did not compromise the innate immune reaction for pathogen clearance.

### IMD suppressed the recruitment of macrophages

Based on the results presented above, IMD increased the number of macrophages in the periphery but did not affect the number in the bone marrow. Thus, IMD may affect the recruitment of macrophages from the bone marrow to the circulation. The MCP-1 (CCL2)/CCR2 system is considered the most important CC chemokine ligand/receptor system involved in regulating the oriented migration and infiltration of monocyte/macrophage to sites of infection^27–29^. IMD did not affect MCP-1 (CCL2) secretion from macrophages (Fig. 4l). We then asked whether IMD affected CCR2 expression in monocytes/macrophages and the subsequent recruitment of macrophages to the periphery.

**Fig. 5:**
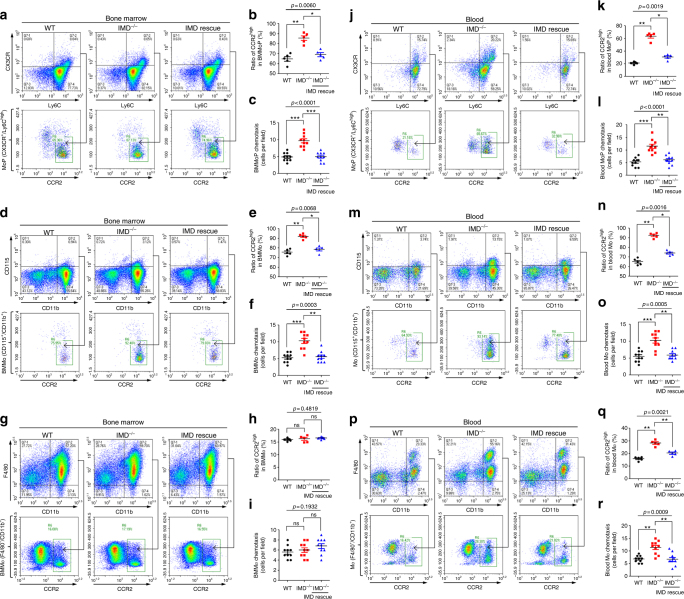
IMD suppressed the recruitment of macrophages from bone marrow to the periphery via decreasing CCR2^high^ cells. The bone marrow samples (**a**, **d**, **g**) and blood samples (**j**, **m**, **p**) from WT, IMD^−/−^, or IMD^−/−^ mice rescued by IMD_40_ injection were analyzed using flow cytometry. Gating strategy: monocyte progenitors (MoP, identified as Ly6C^high^/CX3CR^+^); monocytes (Mo, identified as CD115^+^/CD11b^+^); macrophages (Mϕ, identified as F4-80^+^/CD11b^+^). Ratio of CCR2^high^ (%) in BMMoP (**b**), BMMo (**e**), BMMϕ (**h**), blood MoP (**k**), blood Mo (**n**), and blood Mϕ (**q**) were quantified. The chemotaxis toward MCP-1 (CCL2) of the BMMoP (**c**), BMMo (**f**), BMMϕ (**i**), blood MoP (**l**), blood Mo (**o**), and blood Mϕ (**r**) were tested and quantified. Significance was assessed by Kruskal–Wallis test followed by non-parametric Dunn’s post-hoc analysis

Monocytes were considered the sole precursors of tissue macrophages^30,31^. We examined bone marrow samples from the WT and IMD^−/−^ mice using flow cytometry. Monocyte progenitors in the bone marrow (MoP, identified as Ly6C^high^/CX3CR^+^)^30^ expressing a high level of CCR2 were significantly increased in the IMD^−/−^ mice, and this effect was rescued by IMD administration (Fig. 5a, b). Thus, IMD depletion increased the ratio of CCR2^high^ cells in the MoPs. A similar pattern of CCR2 expression was observed in bone marrow monocytes (BMMo, identified as CD115^+^/CD11b^+^) (Fig. 5d, e). Based on these results, IMD may affect the recruitment of macrophage precursors to the periphery by downregulating CCR2 expression. The altered migration of monocytes was evidenced by the results of the chemotaxis assay, which showed that the IMD deletion significantly increased the migration of bone marrow monocytes and progenitors towards MCP-1, whereas IMD_40_ reduced this directed migration (Fig. 5c, f). However, IMD deletion or IMD peptide rescue did not affect the ratio of CCR2^high^ bone marrow macrophages (BMMϕ, identified as F4-80^+^/CD11b^+^) or their migration (Fig. 5g–i). Therefore, IMD may not directly affect the macrophages in the bone marrow. Monocytes are produced in the bone marrow, from which they migrate to the periphery^32,33^. Thus, IMD may suppress the recruitment of monocytes from bone marrow to the periphery by downregulating CCR2 expression, but it does not affect the already differentiated macrophages in the bone marrow.

We then examined the CCR2 expression profile in circulating monocyte progenitors and monocytes. The ratio of CCR2^high^ cells was significantly increased in the IMD^−/−^ mice, similar to the patterns observed in bone marrow monocytes (Fig. 5j, k, m, n). Interestingly, macrophages (F4-80^low^/CD11b^+^ and F4-80^high^/CD11b^+^) in the periphery also consisted of a higher ratio of CCR2^high^ cells in IMD^−/−^ mice (Fig. 5p, q). IMD deletion increased the migration of circulating monocytes/progenitors and macrophages, whereas IMD_40_ administration decreased migration (Fig. 5i, o, r). The results were consistent with our observation that IMD depletion increased macrophage infiltration in the liver and lungs of CLP-treated mice (Fig. 4m, n). Based on these data, IMD reduces the number of macrophages in the periphery by decreasing the number of CCR2^high^ cells, thus suppressing the recruitment of monocytes from the bone marrow to the circulation and the recruitment of both monocytes and macrophages from the circulation to the sites of infection.

### IMD significantly alleviated acute organ injuries

**Fig. 6:**
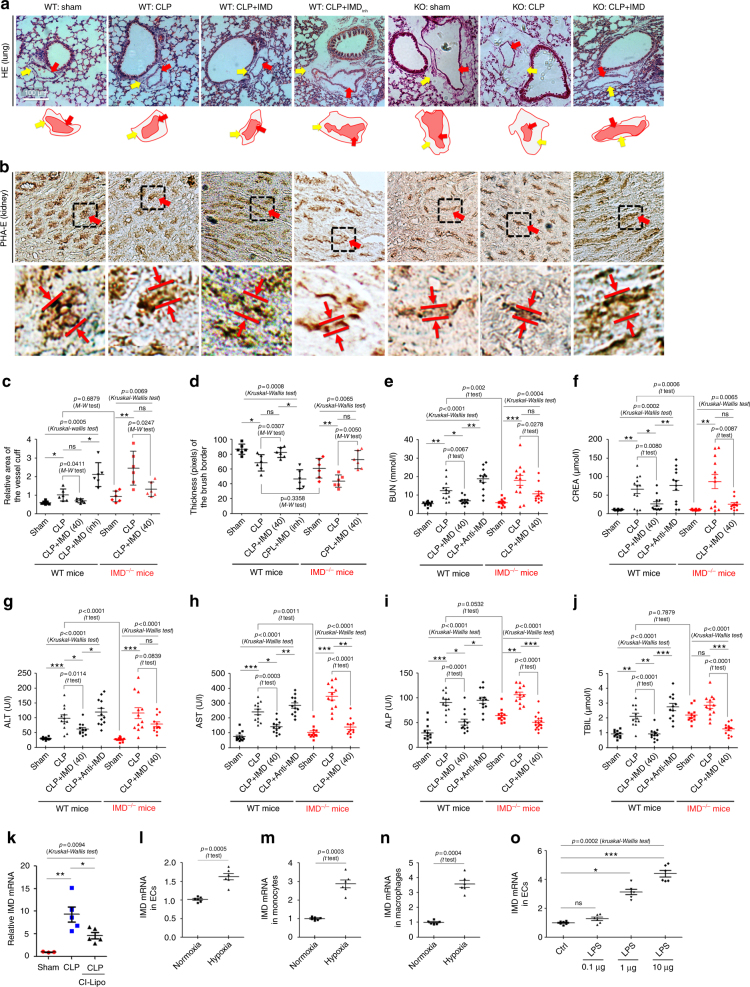
IMD alleviated the lung oedema and acute kidney injuries in sepsis. **a** Representative images show the HE-stained lungs. Lower panels: diagrams of the peri-vascular cuffs. The red arrows indicate the vessel area, and the yellow arrows indicate the cuff area. The ratio of peri-vascular cuff area/vessel area in a randomly chosen field was calculated using ImagePro Plus, and each data (the dot that was shown in the image) represents a mean value of five randomly chosen fields from one mouse, and the statistical data were calculated using six mice (*n* = 6). **b** The PHA-E-stained kidney samples. The red arrows point the brush borders, which indicate the thick microvilli-covered surface of proximal tubules. Lower panels: the measurement of the brush border thickness. **c** The ratio of lung cuffing area referred to the vessel area per lung (**d**) and the thickness of the brush border (pixels) per kidney were measured using Image-pro Plus and presented as plots ± SD. Each dot represents one mean level of five randomly chosen fields from one mouse, and the statistical data were calculated using six mice (*n* = 6). **e–j** After 9 h of CLP surgery, the blood concentration of BUN, CREA, ALT, AST, ALP, and TBIL was measured. Data were presented as scatter plots with mean ± SEM (*n* = 12). **k** The mRNA level of IMD in the liver relative to the control (Sham) were quantified. **l–n** The mRNA level of IMD in HUVEC (**l**), THP-1 (**m**), and peritoneal macrophage (**n**) were incubated in normoxia and hypoxia (1% O_2_). **o** The mRNA level of IMD in HUVECs treated with different doses of LPS. Significance was assessed by Kruskal–Wallis test followed by non-parametric Dunn’s post-hoc analysis (**c–k, o**) and *t* test (**l–n**). The control mice (WT or IMD^−/−^) was compared by Mann–Whitney test (**c, d**) or *t* test (**e–j**) particularly

We hypothesized that IMD may serve as an endogenous stabilizer and a protective factor during sepsis. The widespread perivascular cuffing (Fig. 6a, c) and vacuole degeneration (Supplementary Fig. 12) observed in the lungs and kidneys were histological surrogates of lung oedema and acute kidney injury. IMD_inh_ administration further exacerbated the lung oedema and kidney vacuole degeneration. Interestingly, although IMD^−/−^ mice appeared healthy, the lungs and kidneys from untreated IMD^−/−^ mice showed considerable spontaneous perivascular cuffing and vacuole degeneration. In addition, CLP surgery caused more severe lung oedema and kidney injury in IMD^−/−^ mice than in the WT mice. In contrast, IMD_40_ administration markedly reduced the lung oedema and vacuole degeneration in septic IMD^−/−^ mice and WT mice (Fig. 6a, c; Supplementary Fig. 12).

The *Phaseolus vulgaris* erythroagglutinin (PHA-E) staining of kidney displays the brush border of the proximal tubules, showing the thick microvilli-covered surface for the absorption of substances. In septic animals, the thickness of brush border was markedly decreased. The collapse of brush border indicates the severity of the impaired function of proximal tubules (Fig. 6b, d). IMD_inh_ administration exacerbated the brush border damage. The IMD^−/−^ mice showed significant shrinkage of the brush border, similar to IMD_inh_-treated septic mice. Thus, functional and structural pathogenic changes occur in the kidneys of the apparently healthy IMD^−/−^ mice (Fig. 6b, d). CLP surgery caused more severe brush border shrinkage in IMD^−/−^ mice than in WT mice. IMD_40_ administration rescued the brush border collapse (Fig. 6b, d), indicating that IMD is important for maintaining the brush border integrity in both normal and septic mice. Those histological changes were consistent with the results of blood urea nitrogen (BUN) and creatine (CREA) tests (Fig. 6e, f), suggesting the alleviation of acute kidney injury upon IMD treatment. In addition, IMD_40_ administration reduced alanine transaminase (ALT), aspartate aminotransferase (AST), alkaline phosphatase (ALP), and total bilirubin (TBIL) levels in both WT and IMD^−/−^ septic mice (Fig. 6g–j). Thus, the acute hepatocellular injury was also alleviated in the presence of IMD.

### IMD was a self-protective factor in response to sepsis

Here, we investigated the source of IMD and its induction in response to sepsis. IMD is expressed in ECs and pericytes in the vascular system^14^. In the present study, IMD expression was significantly increased in the major organs, particularly the liver, in response to sepsis (Fig. 1). Thus, parenchymal cells, such as hepatocytes, may be another source of IMD. According to the IHC staining, some hepatocytes showed stronger staining for IMD when mice were septic (Supplementary Fig. 13, yellow arrow). Interestingly, Kupffer cells, which represent the resident macrophages in the liver, showed an even higher staining intensity of IMD in septic animals (Supplementary Fig. 13, red arrow). When monocytes/macrophages were depleted by clodronate liposome injections^34,35^, the increased IMD transcription in the liver was alleviated (Fig. 6k). Based on these results, monocytes/macrophages may be an important source of IMD in local tissues.

We then investigated the mechanism by which IMD expression is induced in response to sepsis. Tissue hypoperfusion and hypoxia are dominant factors that cause multi-organ dysfunctions in sepsis^36^. Hypoxia inducible factor 1 (HIF-1) is considered the most important transcription factor that responds to hypoxia. HIF-1 acts by binding to HIF-responsive elements (HREs) in promoters that contain the sequence “NCGTG”. Four HRE sites are present within the IMD gene promoter, and the transcription of IMD is dose-dependently increased in response to HIF-1α^11^. We tested ECs (HUVECs), monocytes (THP-1), and macrophages (from peritoneal lavage fluid) under hypoxic and normoxic conditions and found that all three cell lines expressed much higher IMD levels under hypoxia than under normoxic conditions (Fig. 6l–n). In addition, LPS induced IMD expression in HUVECs in a dose-dependent manner (Fig. 6o), indicating that circulating endotoxins may directly stimulate IMD secretion by ECs.

### IMD significantly improved the survival of septic mice

**Fig. 7:**
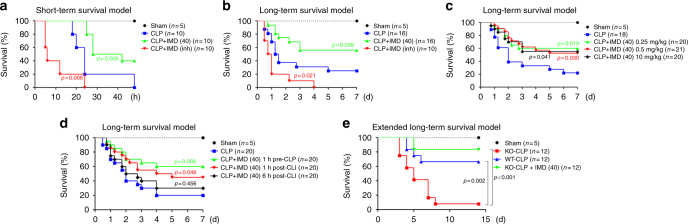
IMD significantly improved the survival of septic mice. **a–e** Kaplan–Meier survival analysis of the septic mice. **a**, **b** The mice were treated with saline, IMD_40_ (0.5 mg/kg) or IMD_inh_ (1 mg/kg) 1 h before CLP surgery (short-term procedure, *n* = 10; or long-term procedure, *n* = 16) and every day for 3 days. **c**, **d** The mice (*n* = 20) received long-term CLP procedure treated with IMD_40_ at different dose (**c**) or timing (**d**). **e** The extended long-term CLP model using WT, IMD^−/−^ mice, and IMD^−/−^ mice (*n* = 12) rescued by IMD_40_ (subcutaneous injection, 0.25 mg/kg/day, 1 h before surgery and every day for 3 days). Each group was compared with the CLP-alone group, and significance was assessed by log rank test

Based on our data, IMD administration may improve the survival rate of septic mice. We tested this hypothesis using the CLP model, the animal model most relevant to clinical sepsis^37^. First, we established a short-term survival CLP model, as described in *Methods*. All mice (*n* = 10) in the CLP-only group died within 48 h after surgery (Fig. 7a). The subcutaneous injection of IMD_40_ (0.5 mg/kg/day) 1 h before surgery significantly improved the survival rate, and 4 of 10 mice still survived after 48 h. IMD_inh_ (1 mg/kg/day), however, worsened the survival outcomes. All mice (*n* = 10) treated with IMD_inh_ died within 24 h (Fig. 7a). The *n*-number in each group is indicated in figure legends.

We then established a long-term CLP model, which better resembles the clinical situation of sepsis. Approximately 20% mice in the CLP-only group survived after 7 days of surgery. Similar to the short-term CLP model, IMD_40_ significantly improved the survival rate (9 of 16 mice were alive after 7 days), whereas all mice treated with IMD_inh_ died within 4 days (Fig. 7b).

We also tested whether the dose and timing of IMD_40_ administration affected the therapeutic effect of IMD on the long-term CLP model. We chose three doses, 0.25, 0.5, and 1.0 mg/kg/day, and treated the septic mice 1 h before surgery. No significant difference in the survival rate was observed among the chosen doses, although the lowest dose, 0.25 mg/kg/day, seemed to yield the best survival outcome (Fig. 7c). On the other hand, the timing of administration was crucial for the therapeutic effect of IMD. The administration of the IMD_40_ injection 1 h before CLP surgery produced the best outcome, with an overall survival rate of approximately 60% (Fig. 7d). The administration of IMD_40_ 1 h after the surgery yielded a reduced survival rate (45%), whereas the IMD_40_ injection 6 h after CLP surgery showed no survival benefit (Fig. 7d).

Finally, we used IMD^−/−^ mice to establish the CLP model. Because the IMD deletion may cause more severe vascular leakage and organ injuries, we postulated that the IMD^−/−^ mice might not tolerate the standard long-term CLP procedure. Therefore, we further shortened the ligation length and reduced the thickness of puncture needle. Seventy-five percent of WT mice (9 of 12) survived 15 days after CLP surgery, whereas only 8.3% IMD^−/−^ mice (1 of 12) survived the surgery (Fig. 7e). The IMD_40_ treatment (0.25 mg/kg/day) significantly improved the survival rate of septic IMD^−/−^ mice to 83% (10 of 12). The data provide strong support for the hypothesis that IMD_40_ may represent a novel sepsis treatment.

### The experimental results correlated with the clinical data

**Fig. 8:**
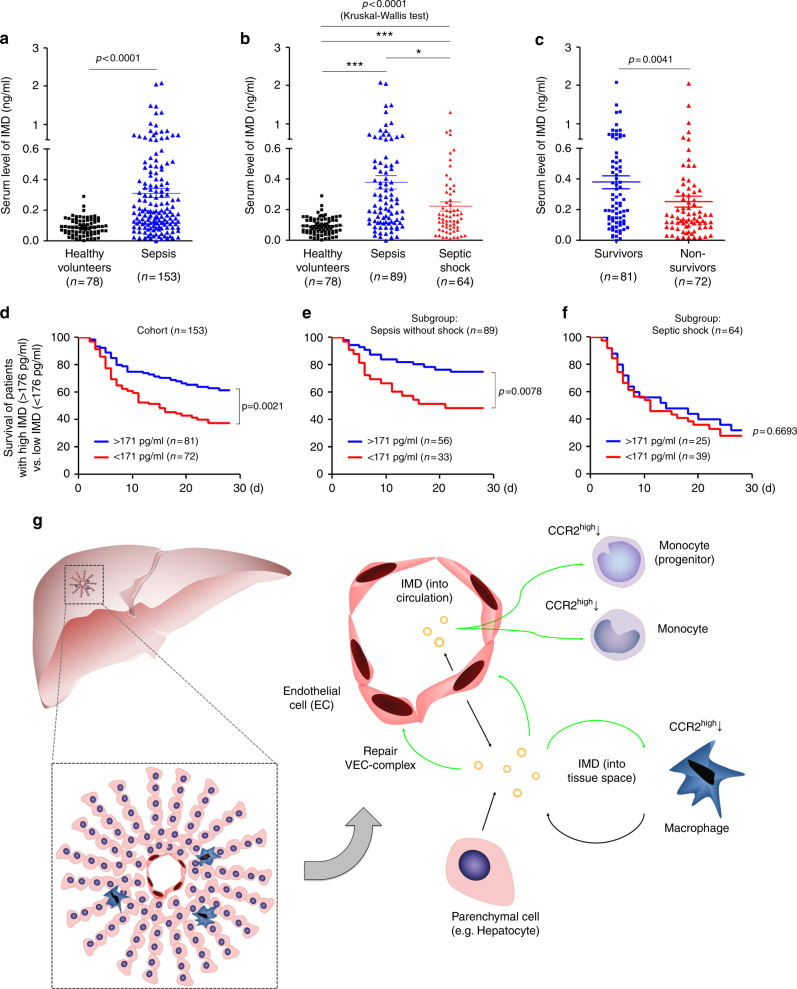
IMD level indicates survival outcome in septic patients. **a** The IMD levels in healthy volunteers and in patients with sepsis were presented as scatter plots with mean ± SEM. **b** The IMD levels in healthy volunteers and in septic patients with or without shock. **c** The IMD levels in the survived and non-survived sepsis patients. **d–f** Kaplan–Meier survival analysis of all patients with sepsis (**d**), sepsis without shock (**e**), and septic shock (**f**) with IMD levels ≥ 171 pg/ml (the median) versus those <171 pg/ml. Significance was assessed by Mann–Whitney test (**a, c**), Kruskal–Wallis test followed by non-parametric Dunn’s post-hoc analysis (**b**), and log rank test (**d–f**). **g** The sources of IMD and its induction in response to sepsis: the circulating IMD in the blood is mainly from the endothelial cells and functions like an endocrine factor. Meanwhile, the parenchymal cells (such as hepatocytes) and infiltrated monocytes/macrophages are the two main sources of IMD in organ tissues, functioning in a paracrine/autocrine manner. During sepsis, the bacterial endotoxin or the widespread hypoxia triggers the transcription of IMD, which in turn repairs the VEC complex through a Rab11-dependent pathway and reduces the secretion of inflammatory factors by downregulating the CCR2^high^ monocytes and macrophages, resulting in self-protective effect. Notably, the IMD peptides released from macrophage themselves can inhibit further infiltration of macrophages into the local tissue by downregulating CCR2, which may represent a feedback loop that maintains the immune balance that was previously disrupted in response to the severe infection

We finally sought to determine whether the experimental findings correlated with the pathogenesis of sepsis in humans. Seventy-eight healthy volunteers and 153 septic patients (89 without shock and 64 with shock) were enroled and classified according to the criteria of the Third International Consensus Definitions for Sepsis and Septic Shock (Sepsis-3)^38^. The patient characteristics are listed in Supplementary Table 1. Serum IMD levels were markedly elevated in septic patients compared with healthy volunteers (Fig. 8a). The change in the IMD level in septic mice was similar (Supplementary Fig. 14). Interestingly, IMD levels at admission were significantly lower in patients with septic shock than in septic patients without shock (Fig. 8b and Supplementary Table 2). We hypothesized that IMD expression might be induced to stabilize the endothelium and ameliorate inflammatory responses during the early phase of sepsis but that IMD would be consumed gradually during disease progression. Thus, a higher level of IMD may be a prognostic factor representing a better outcome. Indeed, a markedly higher IMD level was observed in survivors than in non-survivors (Fig. 8c). The stepwise binary regression analysis showed a significant association between IMD levels and survival outcomes after adjusting for age and sex (Table 1).

**Table 1:** Stepwise binary regression analysis

**Stepwise binary logic regression analysis (survivor/non-survivor)**						
Step 1^a^	*B*	S.E.	Wald	df	Sig.	Exp (*B*)
IMD	−1.800	0.667	7.290	1	0.007**	0.165
Sex	0.557	0.382	1.162	1	0.145	1.745
Age	0.039	0.011	11.663	1	0.001***	1.040
Constant	−2.227	0.762	8.536	1	0.003**	0.108

Adjusting for age and sex, a significant association was observed between IMD levels and survival outcomes

^a^Variable(s) entered on Step 1: IMD, sex, and age; *p < 0.05, **p < 0.01, and ***p < 0.001

In addition, IMD levels higher than the median value (171 pg/ml) were associated with a younger age, lower severity scores, and lower mortality rates (Supplementary Fig. 15 and Supplementary Table 3). According to the Kaplan–Meier survival analysis, patients with IMD levels ≥ 171 pg/ml had a significantly increased probability of survival (Fig. 8d). We further subdivided the patients into early stage (sepsis without shock) and late stage (septic shock). IMD levels were significantly associated with improved survival in patients in the early stage of sepsis (Fig. 8e) but not in patients already presenting shock (Fig. 8f). This finding is consistent with the data presented above showing that earlier IMD administration resulted in a better survival outcome (Fig. 7). Based on the clinical data, IMD has therapeutic potential as a sepsis treatment, and early intervention may be critical to reduce mortality.

## Discussion

Sepsis is now considered a syndrome rather than a disease^39^. Cell death in the heart, kidney, liver, and lung is relatively minor and does not reflect the severity of multi-organ dysfunction^40^. On the other hand, the vascular hyperpermeability and oedema are widespread throughout the body, which exacerbate multi-organ dysfunction. Organ failure is more likely due to a “cell stunning” rather than permanent cell damage^40,41^. Most patients who survive sepsis and display acute renal failure recover baseline renal function, suggesting that excessive cell death does not occur in the kidney during sepsis^42^. Thus, the stabilization of the endothelial barrier may repair the systemic vessel leakage and provide septic patients the opportunity to recover from organ dysfunction caused by oedema and inadequate tissue perfusion.

In the present study, we show that IMD, a natural peptide belonging to the calcitonin family, may be an endogenous protective factor regulating sepsis pathogenesis. IMD markedly reduced systemic hyperpermeability and organ oedema by repairing the endothelial barrier. According to the histological analyses, although the IMD^−/−^ mice without sepsis appeared healthy, they exhibited pathogenic changes in the endothelial barrier in the lung and kidney, and the level was even comparable to that in WT mice with sepsis (Fig. 6a–d). In addition, macrophage infiltration was significantly increased in the liver of IMD^−/−^ mice without sepsis, and the level was similar to that in WT mice with sepsis (Fig. 4m–p). Thus, IMD depletion leads to a state of spontaneous hyperpermeability and increased macrophage infiltration, which may increase the animals’ susceptibility to sepsis. Indeed, the IMD^−/−^ mice subjected to CLP surgery showed much a worse survival rate than the WT mice (8.3% vs. 75.0%). In addition, IMD significantly decreased the systematic inflammatory response by reducing cytokine production and macrophage infiltration. Importantly, IMD did not compromise the innate immune reaction for pathogen clearance. These characteristics of IMD make it an ideal sepsis treatment. The administration of the mature form of IMD significantly improved the survival of septic mice, and the experimental results correlated with the clinical data. Higher IMD levels correlated with a lower severity and greatly improved survival outcomes in patients.

IMD was reported to stabilize the endothelial barrier^11–13^, likely due to the stabilization of VEC complex^12,14^. However, researchers have not identified the mechanism by which IMD regulates VEC activity to maintain the integrity of VEC complex. Here, IMD promoted the relocation of VEC endosomes to cell–cell contacts and filopodia of ECs to re-establish the VEC complex. Rab11, a small GTPase, plays a major role in this process. Thus, IMD dynamically repairs the disrupted endothelial AJs, rather than simply preventing the endothelial AJs from dissociating.

The source of IMD and its induction in response to sepsis must be identified. Considering that 1–6 × 10^13^ ECs with a total surface area of 4000–7000 m^2^ are present throughout the body^43^, ECs may be the main source of IMD in the circulation, which functions as an endocrine-like factor; meanwhile, tissue monocyte/macrophages and some parenchymal cells (such as hepatocytes) may be the main source of IMD in local tissues, and those IMD peptides may function in an autocrine/paracrine manner. Interestingly, the IMD peptides released from macrophages may prevent further infiltration of macrophages into the local tissue by downregulating CCR2, which may represent a feedback loop that maintains the immune balance that was previously disrupted in response to the severe infection (Fig. 8g). The IMD upregulation observed in early stage of sepsis may be a self-protective mechanism against the hyperpermeability and cytokine storm. During sepsis, bacterial endotoxins or widespread hypoxia may trigger the transcription of IMD, which in turn alleviates the vascular leakage and the secretion of inflammatory factors, resulting in self-protective effect. However, as the disease progresses, the secreted IMD will be consumed gradually, and the capacity of the source cells to produce IMD may be weakened because of the increasingly severe multi-organ dysfunction. Consequently, the IMD level may decrease in the late stage of sepsis.

The vasodilatory effect of intravenously injected IMD may worsen the clinical condition of septic patients. In addition, the direct injection of the short peptides into the blood may result in rapid peptide degradation, similar to insulin (contains 51 amino acids), which has a half-life of only 4–6 min if directly injected intravenously. The widely used method for insulin delivery is the subcutaneous (s.c.) injection. We therefore tested whether an s.c. injection of IMD_40_ affected the blood pressure. As shown in Supplementary Fig. 16, an s.c. IMD_40_ (1 mg/kg) injection had little effect on blood pressure. Increasing the doses of IMD_40_ in the s.c. injection to 2.5, 5.0, and 10.0 mg/kg did not affect the blood pressure. The dose of 10.0 mg/kg is 20–40 times higher than the therapeutic dose of IMD_40_. Thus, the s.c. injection of IMD_40_ is a safe delivery method for the treatment of sepsis. The effect of IMD on endothelial barrier function and inflammatory response is relatively slow and persistent. Thus, slowly released under the skin may be a better way to deliver IMD_40_ into the body. The most appropriate administration methods may be s.c. injection or using the micro-injection pump, similar to the way insulin is administered.

Plasma IMD levels were recently shown to be elevated in rats with LPS-induced sepsis^44^, and IMD protected cardiomyocytes in the CLP rat model by regulating calcium channels^45^. Nevertheless, researchers have not determined the role of IMD in the pathogenesis of sepsis. Here, we revealed the mechanism underlying the protective effects of IMD on sepsis, and provided experimental and clinical evidence that the IMD peptide might be a novel therapeutic agent for treating sepsis. The effect of IMD on the endothelium is “dynamic”, because IMD promotes the transport of VEC vesicles back to cell–cell contacts to repair the disrupted VEC complex rather than simply preventing it from dissociating. This effect may make IMD a more suitable treatment to repair the already damaged endothelium in septic patients. In addition, IMD alleviates the “cytokine storm” and suppresses the infiltration of macrophages in sepsis. The “cytokine storm” is a pathophysiological hallmark of sepsis. Thus, the dual roles of IMD to concurrently ameliorate the inflammatory response and repair the inflammatory mediator-induced endothelial disruption may enable it to achieve a better therapeutic efficacy for sepsis treatment.

## Methods

### Cells and reagents

The monocyte cell line THP-1 was obtained from ATCC and routinely cultured in complete RPMI-1640. THP-1 was used to detect the IMD expression in monocyte. HUVECs were isolated from umbilical cords and routinely grown in EGM-2, which was obtained from Lonza (Cat. CC-3162). All cells were tested for mycoplasma contamination. The VEC antibody (Cat. 555661; 1:100 dilution) was obtained from BD Pharmingen. The anti-IMD mAb (clone number: 1106CT1.2.1; isotype: IgG2b; 1:100 dilution) was obtained from Abgent. The Alexa Fluor®488-conjugated secondary antibody (Cat. A21201; donkey anti-mouse, 1:200 dilution) and Alexa Fluor®568-conjugated phalloidin (Cat. A12380; 1:200 dilution) were obtained from Invitrogen. The rabbit anti-IMD polyclonal antibody was customized and purchased from Abgent. IMD_40_ (IMD_8–47,_ the mature form of IMD peptide, containing 40 amino acid residues) and IMD_inh_ (IMD_17–47_, a truncated form of IMD_40_ with 31 amino acids that was used as the competitive inhibitor of IMD^23^) were synthesized and purchased from Shinegene. The concentration of the peptides used in each experiment is indicated in figure legends.

### Real-time RT-PCR

Total RNA was isolated from the heart, liver, spleen, lung, kidney, and intestine of septic mice using Trizol Reagent, which was obtained from Invitrogen. Reverse transcription was performed with a Superscript II Two-Step RT-PCR Kit (Invitrogen). PCR was performed using SYBR® Green PCR Master Mix (Applied Biosystems). Gene expression was normalized to GAPDH. The primers for detecting IMD mRNA were as follows:

Sense: 5′ CGACCCGTCAAACGCATGGAG 3′ and Antisense: 5′ ACAGGCGGTGGCTGAGATTC 3′.

### Immunohistochemical analysis of IMD expression

All animal experiments were approved by the Animal Ethics Committee of Sichuan University and performed according to institutional and national guidelines. Approximately 500 Balb/c or C57/BL6 female mice (8–10 weeks, 20–25 g, housed in specific pathogen-free [SPF] conditions) were used in this study. The septic mice were anaesthetized and perfused transcardially with 4% paraformaldehyde in PBS for 10 min. The heart, liver, spleen, lung, kidney, and intestine were removed, post-fixed with 4% paraformaldehyde for 24 h, embedded in paraffin, and sectioned at a 5-μm thickness. Sections were stained with the anti-IMD pAb (1:200), and signals were developed by incubating the sections with DAB chromogen (brown) and counterstaining with haematoxylin (blue). IMD expression was scored as follows: 0 points, no positive cells; 1 point, <10% positive cells; 2 points, 10–50% positive cells; 3 points, 51–80% positive cells; and 4 points, >80% positive cells. The staining intensity was rated as follows: 1 point, weak staining; 2 points, moderate intensity; and 3 points, strong intensity. Points were added to generate overall scores. The H&E staining was scored by two blinded observers.

### Fluorescent microscopy and quantification

HUVECs treated with VEGF and TNF-α in the presence or absence of IMD_40_ (at the doses indicated in the figure legends) were fixed with 4% PFA, and stained with anti-VEC (1:100) and an Alexa Fluor®488-conjugated secondary antibody (1:200), followed by the staining with Alexaluor®568-conjugated phalloidin (1 μg/ml) and DAPI (20 μg/ml). Fluorescent images were acquired using a Zeiss Z2 microscope. The VEC and F-actin fluorescent signals were measured using Image-Pro Plus ver.5.0.2.9. The quantification method is described below. Step 1, the area of cell–cell contacts was selected, and the intensity of F-actin staining was quantified. Step 2, the intensity of VEC staining in the same area was quantified. Step 3, the ratio of the intensities of VEC/F-actin staining in one field was calculated and expressed as a dot in the statistical graph. The relative intensity of the VEC signal to the F-actin signal at cell–cell contacts was quantified from 10 randomly chosen fields in two experiments (Supplementary Fig. 17).

VEC enrichment in the protrusions that connected two adjacent HUVECs was determined by calculating the intensity of the VEC signal relative to the F-actin signal in the same area of the cell–cell contacts. VEC enrichment in the filopodia was determined by counting the VEC endosomes in the free filopodia extending from the cell surface. The number of filopodia that connected adjacent HUVECs was determined by counting the number of the anastomosed filopodia between two completely separate HUVECs. Ten randomly chosen fields from each image captured in two independent experiments were quantified.

### Miles assay

The in vivo permeability assay has been described^46^. Briefly, EB was injected into the tail veins of mice (6–8 weeks of age), followed by treatment with saline or IMD. After 30 min, VEGF (50 ng in 10 μl of saline) or TNF-α (20 ng in 10 μl of saline) was injected into the dermis to induce permeability. After another 30 min, mice were sacrificed and an 8-mm biopsy located around the injection site (using the site of injection as the centre point for the biopsy) was removed. Permeability was quantitated by monitoring the elution of the EB dye in formamide at 56 °C overnight, and the absorbance was measured at 630 nm. The data were acquired from two skin samples from each mouse, and five mice per group were analyzed.

### Bronchus bronchoalveolar lavage assay

The method was based on previous reports^47,48^. Briefly, bovine serum albumin (BSA) was injected intravenously 90 min prior to the end of the experiment (the endpoint was set to 9 h after CLP surgery). Mice were sacrificed and a bronchoalveolar lavage (BAL) of the right lung was performed twice with 400 μl of saline each. Two hundred fifty microliters were pooled from each BAL aliquot, and BAL and plasma BSA concentrations were determined by ELISA. Permeability was assessed by calculating the BSA/BAL and BSA/plasma ratios. The amount of protein leakage in the lung was consistent with our previous data obtained from the EB leakage assay.

### CLP

In brief, Balb/c or C57/BL6 female mice (8–10 weeks, 20–25 g) were anesthetized with an intraperitoneal (i.p.) injection of pentobarbital sodium (50 mg/kg). An abdominal midline incision was performed and the caecum was isolated and ligated. The ligated caecal stump was then perforated 1–4 times using 18- or 22-gauge needles with or without supportive treatment. The caecum was placed back into its normal intra-abdominal position and the abdomen was closed with running 5–0 prolene sutures. Sham-operated mice underwent identical procedures, except for the CLP.

*Short-term survival CLP model*: The caecum was ligated 7.5 mm from the caecal tip at a site distal to the ileocecal valve, and the ligated caecal stump was then perforated using two “through and through” punctures (18-gauge needle), without supportive treatment.

*Long-term survival CLP model*: The caecum was ligated 7.5 mm from the caecal tip, and the ligated caecal stump was then perforated by one “through and through” puncture (18-gauge needle). Mice received a single intramuscular injection of Ciprofloxacin at a dose of 20 mg/kg and subcutaneous fluid resuscitation with 800 μl of saline immediately after the surgery.

*Extended long-term survival CLP model*: The caecum was ligated 5 mm from the caecal tip, and the ligated caecal stump was then perforated by one “through and through” puncture (22-gauge needle). Mice received a single intramuscular injection of Ciprofloxacin at a dose of 20 mg/kg and subcutaneous fluid resuscitation with 800 μl of saline immediately after the surgery.

The timing and dose of the administration of IMD were determined by s.c. injecting IMD_40_ (0.5 mg/kg/day) or IMD_inh_ (1 mg/kg/day) in 100 μl of saline 1 h before CLP surgery and every day for 3 days after the CLP surgery, unless indicated otherwise. The administrations of IMD and other agents were blinded to the experiment design including groups. Data collection and analysis was not blinded.

*Severe CLP model for EB leakage assay*: The ligation length was elongated (the caecum was ligated 1.2 cm from the tip) and four perforations were induced with a thick needle (18-gauge) to ensure a similar level of vascular leakage as the LPS model. No supportive treatments were administered. Those treatments would cause more severe symptoms than the short-term CLP survival model.

### LPS-induced endotoxaemia

Female Balb/c mice (8–10 weeks, 20–25 g, seven mice in each group) were i.p. injected with a lethal dose of LPS (055:B5, Sigma), 24 mg/kg, by investigators who were blinded to the other treatments^49^. IMD (0.5 mg/kg/day) in 100 μl of saline was s.c. injected 1 h before the LPS injection.

### Antibody feeding assay

HUVECs were plated on coverslips coated with 0.1% gelatine, starved overnight with 0.5% FBS, and labelled with anti-VEC in PBS for 30 min on ice. After labelling, coverslips were washed with PBS, transferred to pre-warmed EGM-2 medium, and stimulated with IMD (2 μM) or vehicle at 37 °C for 10 min. Cells were then washed with mild acid to remove the surface antibodies, fixed with ice-cold acetone for 15 min, and permeabilized with PBS containing 0.1% Triton X-100 for 10 min. Cells were incubated with an Alexa Fluor®488-conjugated secondary antibody for 30 min to detect VEC. After successful staining was confirmed under microscope, cells were incubated with an anti-Rab11 antibody followed an Alexa Fluor®568-conjugated secondary antibody.

### Cytokine measurement using ELISA

Nine hours after the LPS injection or CLP surgery, 20–25 μl of blood samples were collected from tail tip in EDTA tubes to prevent blood clotting. Levels of TNF-α, IL-1β, IL-6, and MCP-1 in serum were measured using ELISA kits (Millipore), according to the manufacturer’s recommendations. Fifty microliters of culture medium from treated macrophages were collected and subjected to ELISAs to examine cytokine production by the isolated macrophages. The peritoneal macrophages were isolated from the peritoneal lavage fluid and purified by magnetic cell sorting using an anti-CD11b antibody.

### Phagocytosis assay

*Escherichia coli* (K-12 strain) BioParticles® conjugated to Alexa Fluor®594 (Invitrogen) were used according to the manufacturer’s instructions. Steady-state peritoneal macrophages were isolated from the peritoneal cavity of WT and IMD^−/−^ mice. Cells were seeded on 24-well plates and incubated for 1 h at 37 °C; the medium was then removed and replaced with medium treated with or without *E. coli* particles (1 mg/ml), and cells were incubated at 37 °C for 2 h. The phagocytic activity was determined by measuring the fluorescent signal within the macrophages using Image-Pro Plus software.

### Blood bacterial load

Blood samples were collected from the septic mice (WT and IMD^−/−^) treated with IMD_40_ or anti-IMD antibodies 9 h after the CLP surgery. Ten microliters from each sample were plated on blood agar plates, and bacterial colonies (CFU) were counted manually.

### Human data

The observational study using the human data for prospective measurements of IMD levels was approved by the ethics committee of West China Second University Hospital, Sichuan University on February 27, 2013. Initially, we enroled 14 patients with SIRS, 64 patients with sepsis, 36 patients with severe sepsis, and 72 patients with septic shock classified according to the criteria of the International Sepsis Definitions Conference 2001 (Sepsis-2)^50^. In 2016, the Third International Consensus Definitions for Sepsis and Septic Shock (Sepsis-3) was convened^38^ because considerable advances have been made in the pathobiology, management, and epidemiology of sepsis since 2001. In this meeting, the task force concluded that the SIRS criteria lacked specificity and sensitivity and were misleading, and the term “severe sepsis” was redundant. We revised our inclusion criteria accordingly. Fourteen patients with SIRS were excluded. Thirty-six patients with severe sepsis were included in the sepsis group, in which organ dysfunction was represented by an increase in the SOFA score of 2 points or more. Patients without organ dysfunction were excluded. The informed consent was obtained from all subjects.

Seventy-eight healthy volunteers and 153 patients (89 with sepsis and 64 with septic shock) with sepsis were enroled and classified according to the Sepsis-3 criteria (in brief, “SIRS” and “Severe sepsis” were removed, and the patients were classified as “Sepsis without shock” and “Septic shock”)^38^. The cohort was predominantly male (68.6%) and had a median age of 64 years (range 18–90 years). Eighty-nine cases of sepsis without shock (58.2%) and 64 cases of septic shock (41.8%) were included. The main infections were pneumonia (51.6%) and peritonitis (23.5%). Approximately 50.1% of the patients exhibited multiple organ failure (≥2 organs), and 49.9% exhibited single organ failure. The severity scores recorded in the present study were APACHE II with 26.9 ± 8.8 points, SOFA with 10.4 ± 5.1 points, and GCS with 8.1 ± 3.2 points. The 28-day mortality rate was 49.7%.

Blood samples were collected within 24 h of admission to the ICU. After blood collection, serum was separated by centrifugation (2000*g*, 20 min, 4 °C) and stored at −80 °C until ELISA detection. IMD levels in human serum samples were measured using an ELISA kit (Phoenix Pharmaceuticals, Beijing, China). Serum IMD levels are presented as scatter plots showing the mean ± SEM. A Kaplan–Meier analysis showing the survival of patients with IMD levels ≥ 171 pg/ml (the median value of all measurements) compared with the survival of patients with IMD levels < 171 pg/ml is presented.

### Statistics

The statistical power was calculated to determine the *n*-number of each group. For example, the calculation of the statistical power showed that the number of mice used in AST, ALT, ALP, and TBIL testing should at least be 9, 6, 5, and 11, respectively. Thus, the *n*-number of each group was set to 12. No randomization was applied because all mice used were genetically defined, inbred mice.

When comparing two groups for which a Gaussian distribution was not assumed, the unpaired, 2-tailed nonparametric Mann–Whitney *U* test was used; when a Gaussian distribution was assumed, the unpaired, 2-tailed parametric *t* test with Welch’s correction was used. Data from multiple groups were compared using one-way ANOVA (Kruskal–Wallis test) followed by non-parametric Dunn’s post hoc analysis. A *p* value < 0.05 was considered statistically significant. *p < 0.05, **p < 0.01, and ***p < 0.001.

Survival outcomes were analyzed using Kaplan–Meier survival curves, and the significance of the differences was assessed using the log-rank test. For analyses of human data, a cut-off value for the IMD level (171 pg/ml) was defined as the value with the maximum specificity and sensitivity to discriminate between survivors and non-survivors. The correlation between IMD levels and APACHE II and SOFA scores were assessed using Spearman’s correlation coefficients. A further analysis compared the survival of patients with IMD levels ≥ 171 pg/ml with the survival of patients presenting IMD levels < 171 pg/ml (the median value of all measurements). The log-rank test was used to compare the two groups. *p* Values of 0.05 or less were considered to denote a significant difference.

### Data availability

The data that support the findings of this study are available from the corresponding author upon reasonable request. The authors declare that the data supporting the findings of this study are available within the paper and its supplementary information files.

## Electronic supplementary material

Supplementary Information

## References

[1] Riedemann NC, Guo RF, Ward PA (2003). Novel strategies for the treatment of sepsis. Nat. Med..

[2] Russell JA (2006). Management of sepsis. N. Engl. J. Med..

[3] Deutschman CS, Tracey KJ (2014). Sepsis: current dogma and new perspectives. Immunity.

[4] Spicer A, Calfee CS (2012). Fixing the leak: targeting the vascular endothelium in sepsis. Crit. Care.

[5] Lee WL, Slutsky AS (2010). Sepsis and endothelial permeability. N. Engl. J. Med..

[6] Aird WC (2003). The role of the endothelium in severe sepsis and multiple organ dysfunction syndrome. Blood.

[7] Dejana E, Orsenigo F, Lampugnani MG (2008). The role of adherens junctions and VE-cadherin in the control of vascular permeability. J. Cell Sci..

[8] Harris ES, Nelson WJ (2010). VE-cadherin: at the front, center, and sides of endothelial cell organization and function. Curr. Opin. Cell Biol..

[9] Roh J, Chang CL, Bhalla A, Klein C, Hsu SY (2004). Intermedin is a calcitonin/calcitonin gene-related peptide family peptide acting through the calcitonin receptor-like receptor/receptor activity-modifying protein receptor complexes. J. Biol. Chem..

[10] Takei Y (2004). Identification of novel adrenomedullin in mammals: a potent cardiovascular and renal regulator. FEBS Lett..

[11] Pfeil U (2009). Intermedin/adrenomedullin-2 is a hypoxia-induced endothelial peptide that stabilizes pulmonary microvascular permeability. Am. J. Physiol. Lung Cell. Mol. Physiol..

[12] Aslam M (2012). Intermedin (adrenomedullin2) stabilizes the endothelial barrier and antagonizes thrombin-induced barrier failure in endothelial cell monolayers. Br. J. Pharmacol..

[13] Muller-Redetzky HC (2012). Intermedin stabilized endothelial barrier function and attenuated ventilator-induced lung injury in mice. PLoS One.

[14] Zhang W (2012). Intermedin: a novel regulator for vascular remodeling and tumor vessel normalization by regulating vascular endothelial-cadherin and extracellular signal-regulated kinase. Arterioscler. Thromb. Vasc. Biol..

[15] Wang Y (2016). Intermedin ameliorates IgA nephropathy by inhibition of oxidative stress and inflammation. Clin. Exp. Med..

[16] Pang Y (2016). Intermedin restores hyperhomocysteinemia-induced macrophage polarization and improves insulin resistance in mice. J. Biol. Chem..

[17] Sawant DA (2013). Regulation of tumor necrosis factor-alpha-induced microvascular endothelial cell hyperpermeability by recombinant B-cell lymphoma-extra large. J. Surg. Res..

[18] Petrache I, Birukova A, Ramirez SI, Garcia JG, Verin AD (2003). The role of the microtubules in tumor necrosis factor-alpha-induced endothelial cell permeability. Am. J. Respir. Cell Mol. Biol..

[19] Petrache I (2001). Differential effect of MLC kinase in TNF-alpha-induced endothelial cell apoptosis and barrier dysfunction. Am. J. Physiol. Lung Cell. Mol. Physiol..

[20] Shapiro NI, Aird WC (2011). Sepsis and the broken endothelium. Crit. Care.

[21] van der Flier M (2005). Plasma vascular endothelial growth factor in severe sepsis. Shock.

[22] Pickkers P (2005). Vascular endothelial growth factor is increased during the first 48 h of human septic shock and correlates with vascular permeability. Shock.

[23] Roh J, Chang CL, Bhalla A, Klein C, Hsu SY (2004). Intermedin is a calcitonin/calcitonin gene-related peptide family peptide acting through the calcitonin receptor-like receptor/receptor activity-modifying protein receptor complexes. J. Biol. Chem..

[24] Lock JG, Stow JL (2005). Rab11 in recycling endosomes regulates the sorting and basolateral transport of E-cadherin. Mol. Biol. Cell.

[25] Grant BD, Donaldson JG (2009). Pathways and mechanisms of endocytic recycling. Nat. Rev. Mol. Cell Biol..

[26] Gavard J, Gutkind JS (2006). VEGF controls endothelial-cell permeability by promoting the beta-arrestin-dependent endocytosis of VE-cadherin. Nat. Cell Biol..

[27] Serbina NV, Pamer EG (2006). Monocyte emigration from bone marrow during bacterial infection requires signals mediated by chemokine receptor CCR2. Nat. Immunol..

[28] Kuziel WA (1997). Severe reduction in leukocyte adhesion and monocyte extravasation in mice deficient in CC chemokine receptor 2. Proc. Natl. Acad. Sci. U.S.A..

[29] Boring L (1997). Impaired monocyte migration and reduced type 1 (Th1) cytokine responses in C–C chemokine receptor 2 knockout mice. J. Clin. Invest..

[30] Hettinger J (2013). Origin of monocytes and macrophages in a committed progenitor. Nat. Immunol..

[31] van Furth R, Cohn ZA (1968). The origin and kinetics of mononuclear phagocytes. J. Exp. Med..

[32] Swirski FK (2009). Identification of splenic reservoir monocytes and their deployment to inflammatory sites. Science.

[33] Auffray C, Sieweke MH, Geissmann F (2009). Blood monocytes: development, heterogeneity, and relationship with dendritic cells. Annu. Rev. Immunol..

[34] Weber GF (2015). Interleukin-3 amplifies acute inflammation and is a potential therapeutic target in sepsis. Science.

[35] Nemeth K (2009). Bone marrow stromal cells attenuate sepsis via prostaglandin E(2)-dependent reprogramming of host macrophages to increase their interleukin-10 production. Nat. Med..

[36] Cohen J (2002). The immunopathogenesis of sepsis. Nature.

[37] Hubbard WJ (2005). Cecal ligation and puncture. Shock.

[38] Singer M (2016). The Third International Consensus Definitions for Sepsis and Septic Shock (Sepsis-3). JAMA.

[39] Vincent JL, Opal SM, Marshall JC, Tracey KJ (2013). Sepsis definitions: time for change. Lancet.

[40] Hotchkiss RS, Karl IE (2003). The pathophysiology and treatment of sepsis. N. Engl. J. Med..

[41] Sawyer DB, Loscalzo J (2002). Myocardial hibernation: restorative or preterminal sleep?. Circulation.

[42] Wheeler AP, Bernard GR (1999). Treating patients with severe sepsis. N. Engl. J. Med..

[43] Yau JW, Teoh H, Verma S (2015). Endothelial cell control of thrombosis. BMC Cardiovasc. Disord..

[44] Yang SX, Chen YX, Xu J, Yang ZH (2016). Plasma intermedin level indicates severity and treatment efficacy of septic shock in Sprague-Dawley (SD) rats. Med. Sci. Monit..

[45] Zhu Y (2016). Beneficial effect of intermedin 1–53 in septic shock rats: contributions of Rho kinase and BKCA pathway-mediated improvement in cardiac function. Shock.

[46] Jones CA (2008). Robo4 stabilizes the vascular network by inhibiting pathologic angiogenesis and endothelial hyperpermeability. Nat. Med..

[47] Muller-Redetzky HC (2014). Mechanical ventilation drives pneumococcal pneumonia into lung injury and sepsis in mice: protection by adrenomedullin. Crit. Care.

[48] Muller HC (2010). Simvastatin attenuates ventilator-induced lung injury in mice. Crit. Care.

[49] Puneet P (2010). SphK1 regulates proinflammatory responses associated with endotoxin and polymicrobial sepsis. Science.

[50] Levy MM (2003). 2001 SCCM/ESICM/ACCP/ATS/SIS International Sepsis Definitions Conference. Crit. Care Med..

