# Supplementary material for: Intermedin protects against sepsis by concurrently re-establishing the endothelial barrier and alleviating inflammatory responses
Source: Nat Commun. 2018 Jul 6;9:2644. doi: 10.1038/s41467-018-05062-2 (PMC6035189; doi:10.1038/s41467-018-05062-2)
Supplement: Supplementary file 1 — Supplementary Information [file 41467_2018_5062_MOESM1_ESM.pdf]

Title: Intermedin protects against sepsis by concurrently re-establishing the endothelial barrier and alleviating inflammatory responses

Author: Xiao et al.

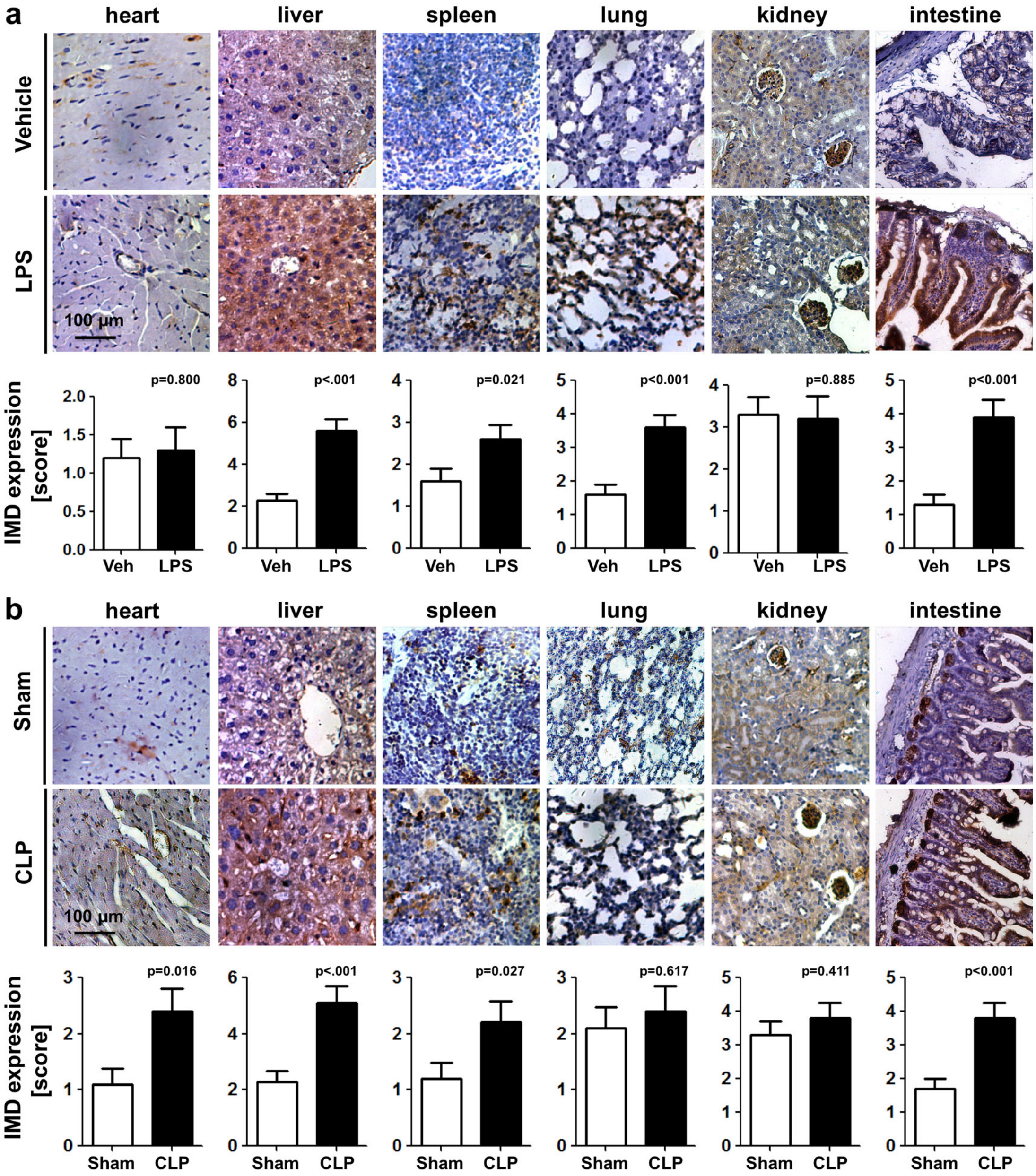

Supplementary Figure 1. Immunohistochemical staining showed high expression of IMD in organs of septic mice. (**a, b**) Organ samples were immunostained for IMD (brown) and counterstained with hematoxylin (blue). The scores of IMD expression were quantified using 25 randomly chosen fields from 5 mice of each group as described in the *Methods*. Data was presented as columns with mean ± SD relative to the vehicle group (the mean level of vehicle group was set to 1.0, n=5). Significance was assessed by Mann-Whitney test.

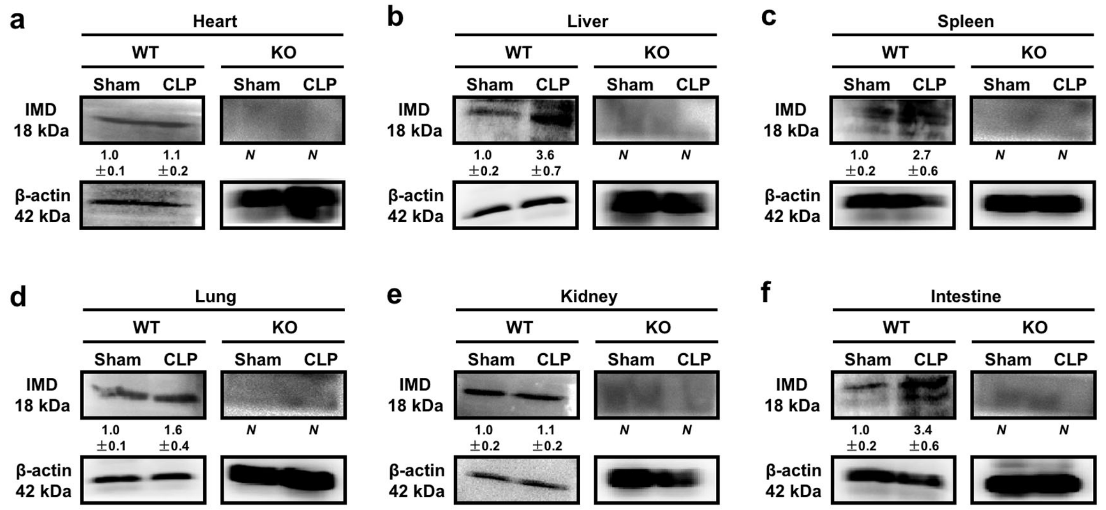

Supplementary Figure 2. Western blot analysis showed the expression of IMD in organs from WT and IMD$^{-/-}$ mice. (**a**) Heart; (**b**) Liver; (**c**) Spleen; (**d**) Lung; (**e**) Kidney; (**f**) Intestine. Data was presented below the blots with mean ± SD relative to the vehicle group (the mean level of vehicle group was set to 1.0, n=5).

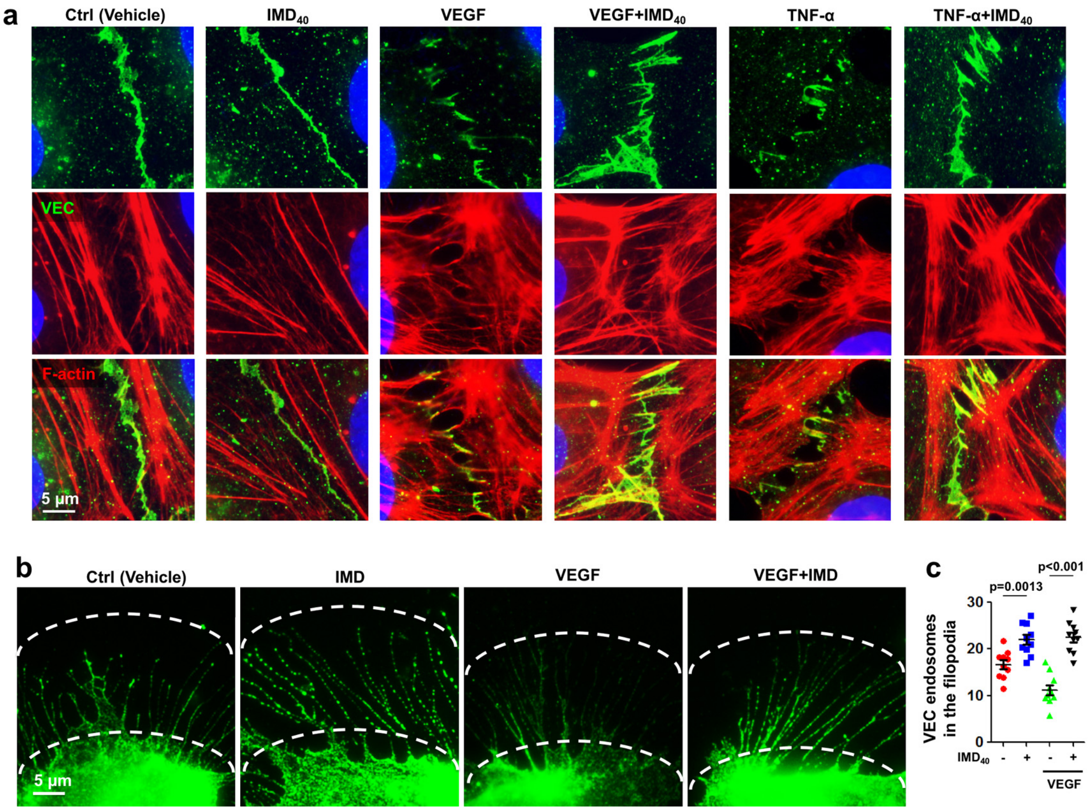

Supplementary Figure 3. IMD promoted VEC accumulating in the cell-cell contact and the free filopodia. (a) The HUVEC monolayer was treated with vehicle (PBS), IMD_{40} (2μM), VEGF (50ng/ml), TNF-α (20ng/ml) alone, or treated with VEGF or TNF-α for 2h followed by treatment of IMD_{40}. Representative images showed the staining of VEC (green) and F-actin (red). (**b**) The sparse HUVECs (not contacted with adjacent cells) treated with VEGF or IMD_{40} were stained for VEC. The green dots indicate the endosomes of VEC in the free filopodia. (**c**) The number of VEC endosomes in the free filopodia outside the cell surface was quantified using 10 randomly chosen fields from 2 experiments.

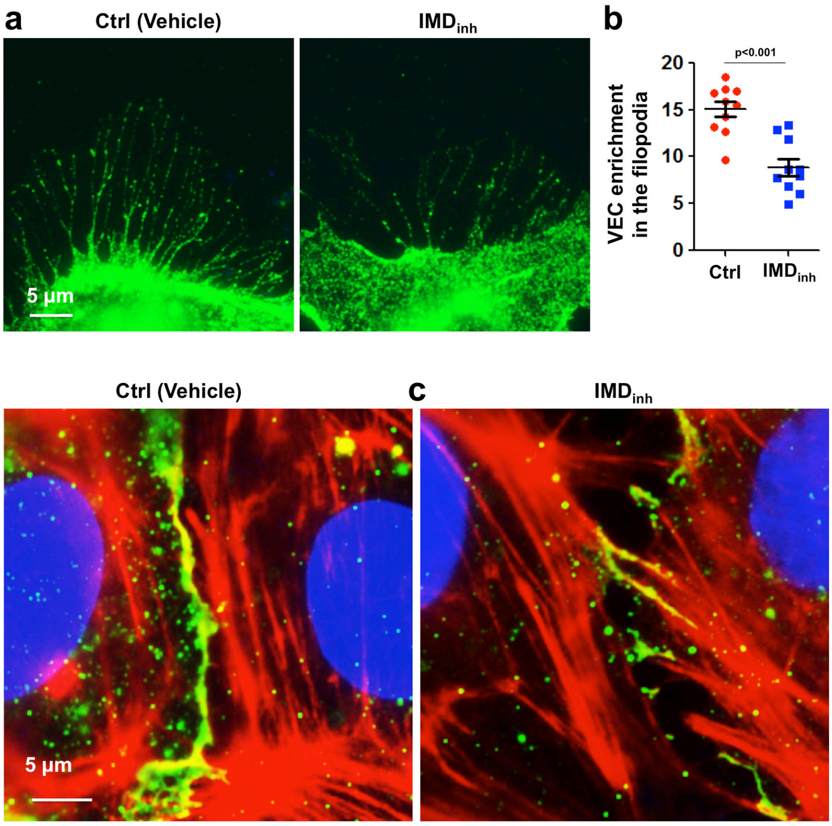

Supplementary Figure 4. Inhibition of IMD reduced the VEC enrichment in the filopodia and cell-cell contact. (**a**) The sparse HUVECs that didn't contact with each other were treated with vehicle or IMD_{inh} were stained for VEC. The green dots indicate the endosomes of VEC in the free filopodia. (**b**) The number of VEC endosomes in the free filopodia outside the cell surface was quantified using 10 randomly chosen fields (from 2 experiments). Data were presented as scatter plots with mean ± SEM. (**c**) The representative images showed that IMD_{inh} disrupted the established VEC-complex.

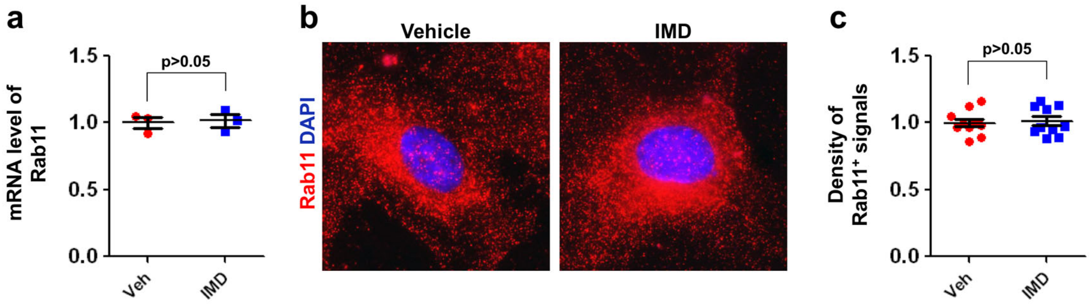

Supplementary Figure 5. Rab11 expression in the HUVECs. (a) The mRNA level of IMD in HUVECs treated with vehicle of IMD_{40}. (**b**) Fluorescent staining of Rab11 of HUVECs treated with vehicle and IMD_{40}. (**c**) The density of $Rab11^{+}$ signals in HUVECs treated with vehicle and IMD_{40} were quantified using 10 randomly chosen fields from 2 experiments. Significance was assessed by *Mann-Whitney test*.

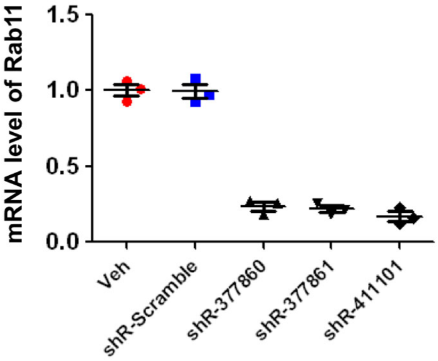

Supplementary Figure 6. The HUVECs were transfected with the shRNA candidates of Rab11 (using the lentiviral vector). The mRNA level of Rab11 was measured by Real-time RT-PCR.

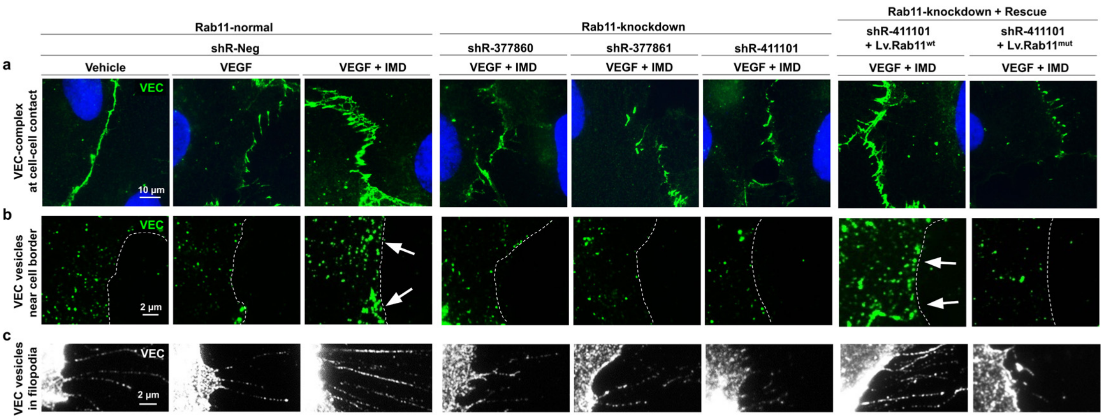

Supplementary Figure 7. Silencing Rab11 abrogated the ability of IMD to repair VEC complex and VEC vesicle transportation (**a, b, c**) The HUVECs were transfected with the shRNAs of Rab11 (shR-377860, shR-377861, shR-411101), followed by the treatment of VEGF with or without IMD_{40}. shR-411101 that targets the 3'-UTR of Rab11 was rescued by transfecting the vector that expresses wild type or mutant Rab11 lacking 3'-UTR. The representative images showing (**a**) the VEC staining intensity at the cell-cell contact, (**b**) the VEC^+ vesicles at the cell border, and (**c**) the VEC^+ vesicles accumulated in the filopodia.

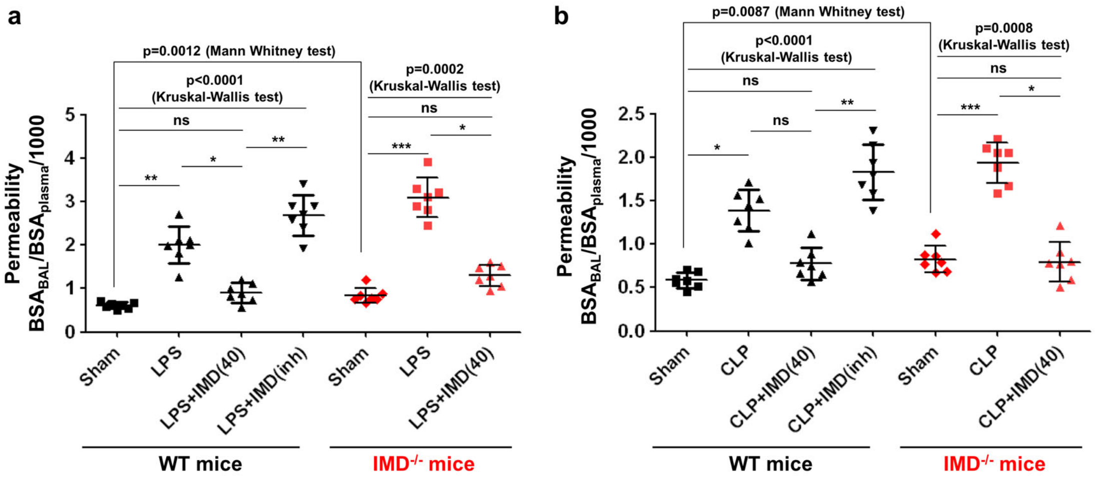

Supplementary Figure 8. Bronchus bronchoalveolar lavage assay to examine the permeability of the lung. WT or $IMD^{-/-}$ mice were injected IMD_{40} (0.5mg/kg) or IMD_{inh} (1mg/kg) subcutaneously 1h before LPS administration (24mg/kg) (**a**) or CLP surgery (**b**), and subjected to the Bronchus bronchoalveolar lavage assay. Data was presented as scatter plots with mean ± SD (n=7 per group). Significance was assessed by *Kruskal-Wallis test* followed by *non-parametric Dunn's post-hoc analysis*.

The control mice (WT or IMD$^{-/-}$) was compared by *Mann-Whitney test* separately.

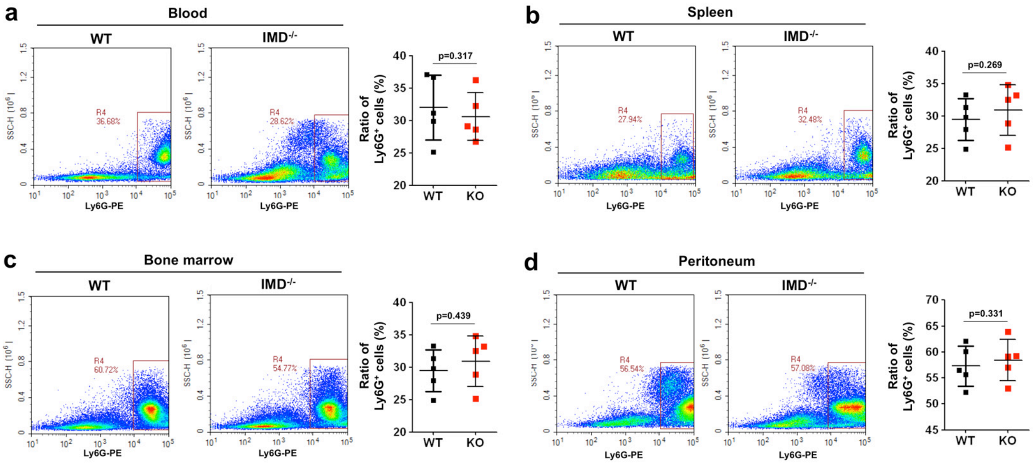

Supplementary Figure 9. IMD did not affect the neutrophil profiles on steady state. The neutrophils were stained using Ly6G. The neutrophil profiles (%) on steady state of blood, spleen, bone marrow and peritoneum were presented as scatter plots with mean ± SEM (n=5 mice).

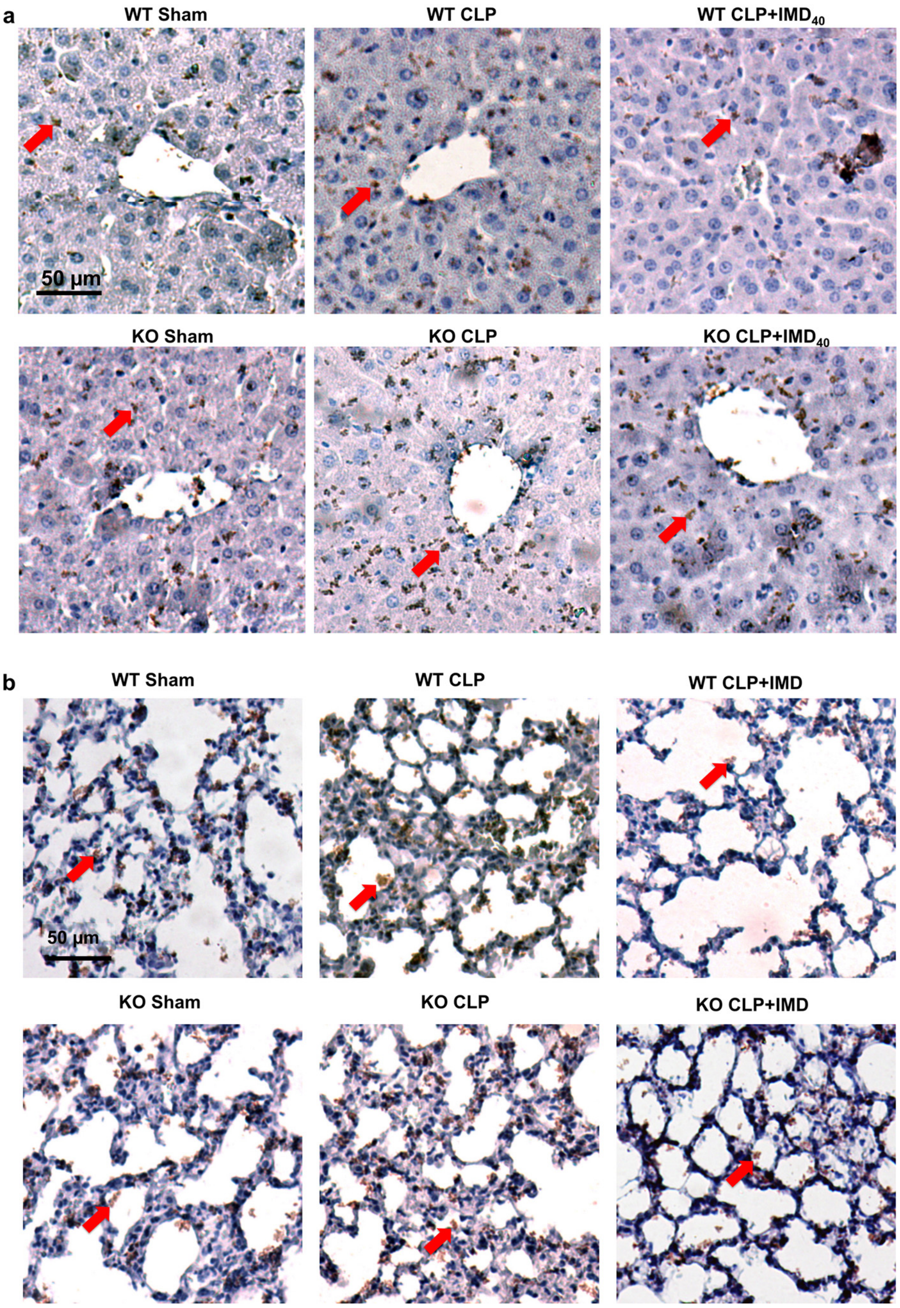

Supplementary Figure 10. The macrophage infiltration in liver and lungs. Nine hours after the CLP surgery, the liver (**a**) and lungs (**b**) were isolated and stained with anti-F4/80. Arrows point the F4/80-positive macrophage.

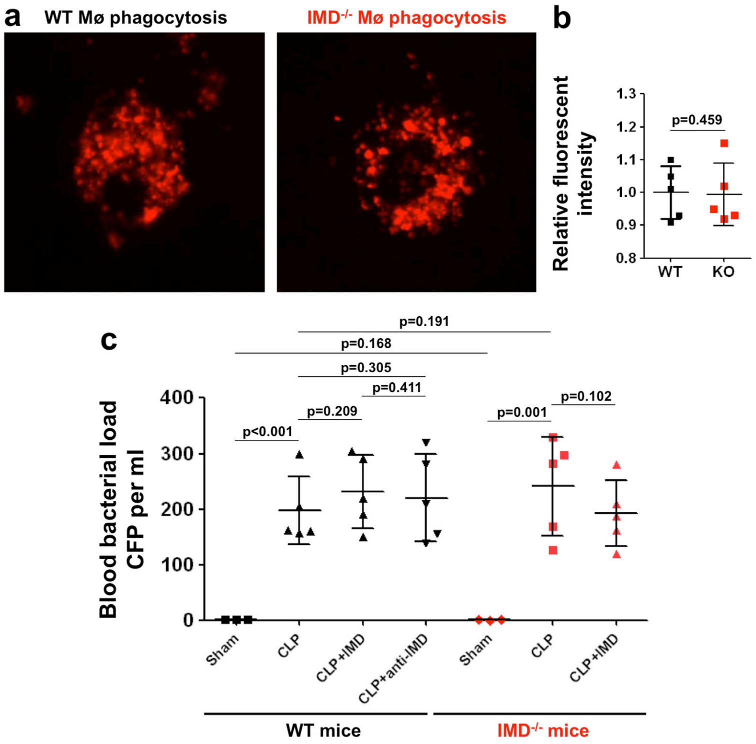

Supplementary Figure 11. IMD did not affect the phagocytic capacity of macrophages and the microbial clearance in the circulation. (**a**) The Phagocytosis assay using the peritoneal macrophages (Mø) isolated from WT or IMD$^{-/-}$ mice. (**b**) The phagocytic capacity was measured by counting the fluorescent signals within the cell using the software Image-pro Plus. The relative fluorescent signals were referred to the WT group (the mean level of WT group was set to 1.0). Data were presented as plots ± S.D. Each dot represents 1 mean level of 10 randomly chosen fields from 1 mouse, and the statistical data were calculated using 5 mice (n=5). (**c**) The colony forming unit (CFU) per ml of the septic mice (WT and IMD$^{-/-}$) treated with IMD_{40} or anti-IMD antibodies. Data were presented as plots ± S.D. (in sham groups, n=3; in other groups, n=5)

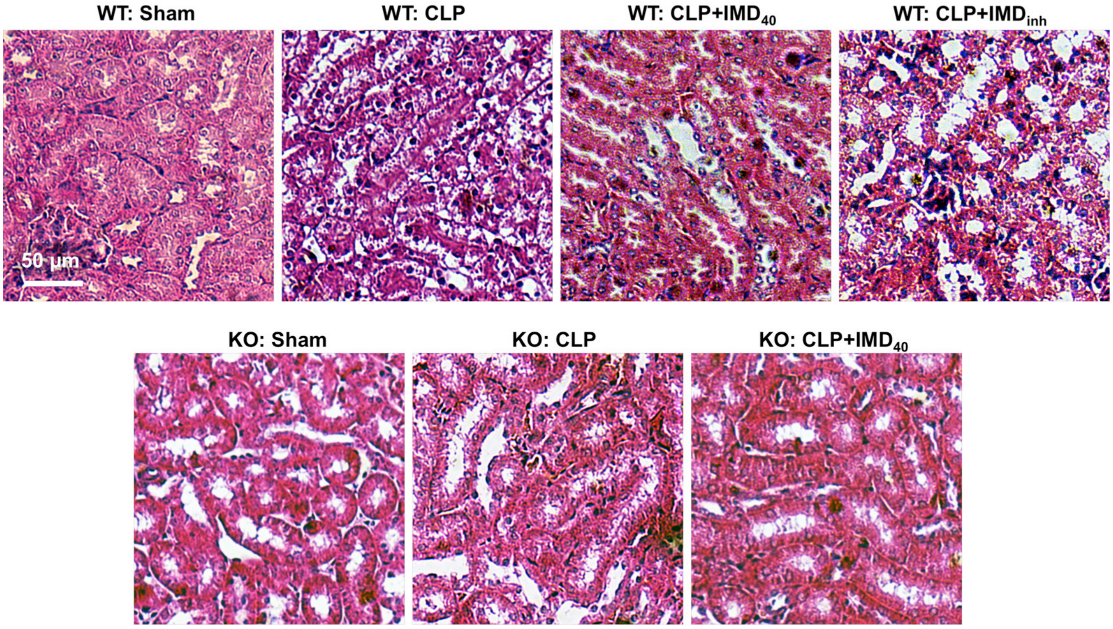

Supplementary Figure 12. IMD alleviated the vacuole degeneration of kidney in sepsis. The representative images of the HE-stained kidney samples showed the vacolation and feather-like structures, which indicate the vacuole degeneration of tubular epithelial cells.

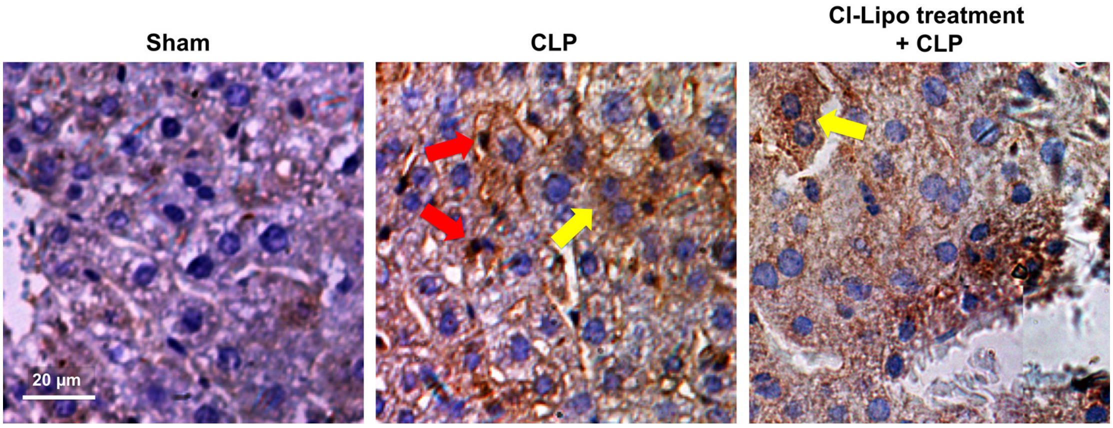

Supplementary Figure 13. IMD expression in the liver tissues of septic mice. The liver sections were stained for IMD, and the hepatocytes (yellow arrows) and kupffer cells (red arrows) showed stronger staining of IMD when mice were in states of sepsis. When the macrophages were depleted by the treatment of clodronate liposome, the expression of IMD was down-regulated (right panel).

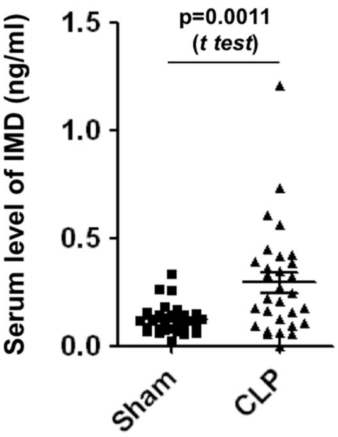

Supplementary Figure 14. The IMD levels in mice received sham-surgery or CLP-surgery were measured by ELISA. Data was presented as scatter plots with mean ± SEM. Significance was assessed by *2-tailed parametric t tests with Welch's correction*.

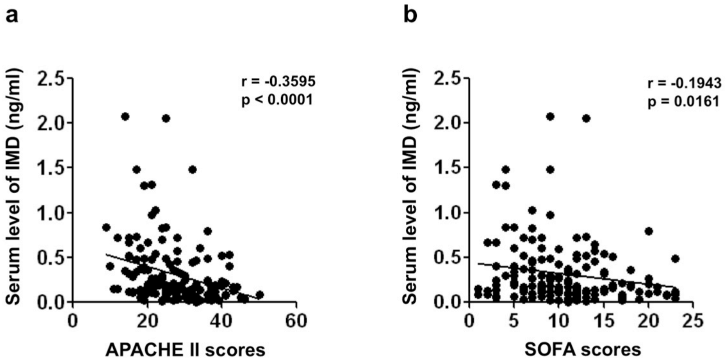

Supplementary Figure 15. Spearman's correlation analysis showed a tendency towards a negative correlation between IMD levels and APACHE II (**a**) and SOFA (**b**) scores in sepsis patients.

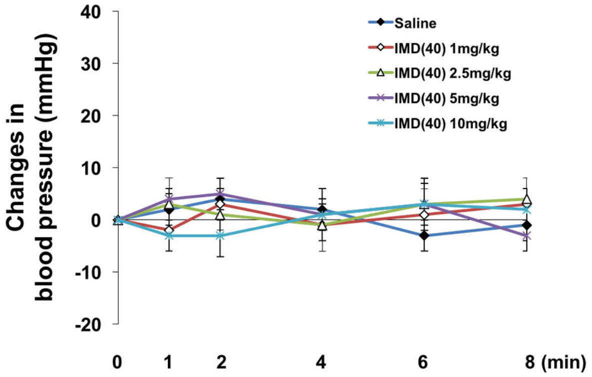

Supplementary Figure 16. The plastic catheter was placed into the carotid artery of the mouse, and the data of the arterial blood pressure was acquired using the multiple channel data acquisition system. Different doses of IMD_{40} were injected subcutaneously (s.c.) into the mice, and the blood pressure was measured.

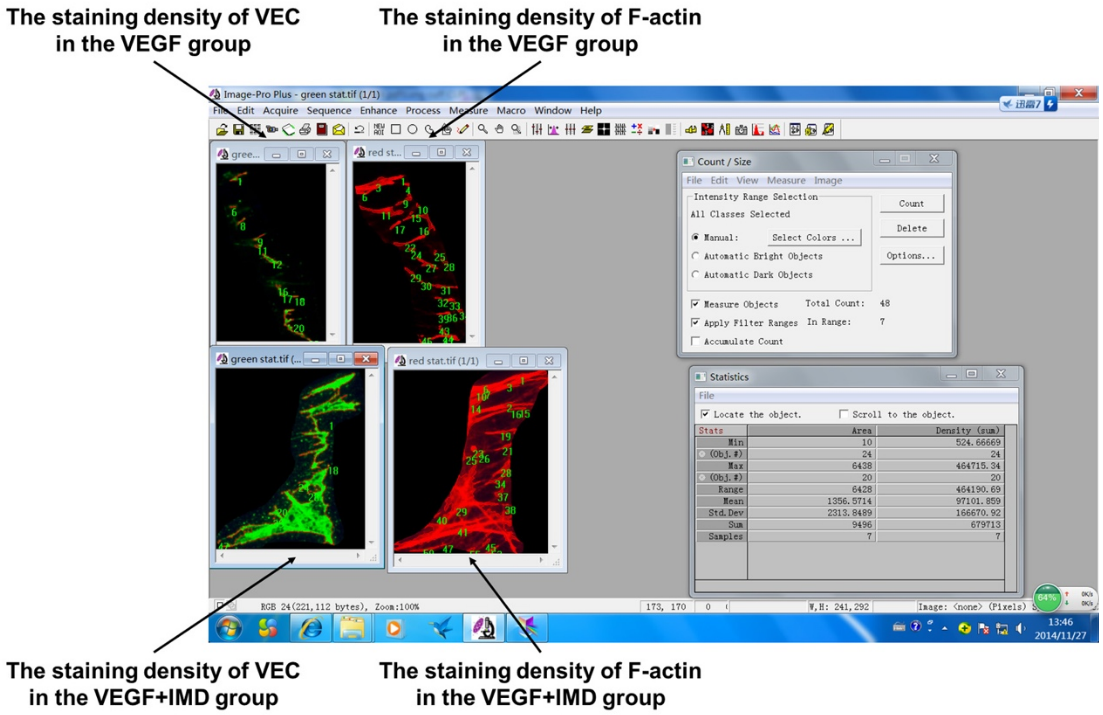

Supplementary Figure 17. The quantification method for the immunofluorescent signal. The VEC and F-actin fluorescent signals were measured using Image-Pro Plus ver.5.0.2.9, the quantification method was as follows: Step 1, the area of the cell-cell contact was chosen, and the staining density of F-actin was quantified; Step 2, the staining density of VEC in the same area was quantified; Step 3, the ratio of the staining density of VEC/F-actin from one field was calculated and expressed as a dot in the

statistical graph. The relative density of VEC signal referred to F-actin at the cell-cell contact was quantified using 10 randomly chosen fields from 2 experiments.

Patients with sepsis (n=153)	
**Demographic data**	
Age, median years (range)	64 (18~90)
Sex, male	105 (68.6%)
**Sepsis characteristics**	
Sepsis (without shock)	89 (58.2%)
Septic shock	64 (41.8%)
**Infections**	
Pneumonia	79 (51.6%)
Peritonitis	36 (23.5%)
Gastrointestinal infection	6 (3.9%)
Wound infection	5 (3.3%)
Meningitis	5 (3.3%)
Endocarditis	2 (1.3%)
Other	23 (15.0%)
**Septic organ failure**	
Multi-organ failure (≥2 organs)	78 (50.9%)

Single organ failure		75 (49.1%)
	RF	28 (18.3)
	ARDS	9 (5.9%)
	ARF	10 (6.5%)
	Other	28 (16.9%)
**Disease Severity Scoring**		
APACHE II (Mean ± SD)		26.9 ± 8.8
SOFA (Mean ± SD)		10.4 ± 5.1
GCS (Mean ± SD)		8.1 ± 3.2
**Outcome**		
Survivor (28 d)		77 (50.3%)
Data presented by number (%) or means ± SD. Abbreviations: RF, respiratory failure; ARDS, acute respiratory distress syndrome; ARF, acute renal failure; ALF, acute liver failure; AHF, acute heart failure; APACHE II, acute physiology and chronic health evaluation II; SOFA, sequential organ failure assessment; GCS, Glasgow coma scale		

Supplementary Table 1. The patient characteristics.

Stepwise binary logic regression analysis (sepsis without shock / sepsis with shock)							
		B	S.E.	Wald	df	Sig.	Exp (B)
step 1[a]	IMD	-1.813	0.704	6.642	1	0.010 **	0.163
	Sex	-0.394	0.365	1.162	1	0.281	0.674
	Age	0.003	0.010	0.075	1	0.785	1.003
	Constant	0.284	0.702	0.164	1	0.686	1.328
a: variable(s) entered on step 1: IMD, Sex, Age							

Supplementary Table 2. Stepwise binary regression analysis. Adjusting for age and sex, a significant association was observed between IMD levels and the survival rate.

Patients' characteristics separated by IMD levels (cut-off value: 171pg/ml) (n=153)			
	IMD≥171pg/ml (n=81)	IMD<171pg/ml (n=72)	p value
**Demographic data**			
Age, median years (range)	59 (18~90)	68 (25~86)	0.0174 *
Sex, male	55 (67.9%)	50 (69.4%)	0.839
**Sepsis characteristics**			
Sepsis (without shock)	56 (69.1%)	33 (45.8%)	0.0037 **
Septic shock	25 (30.9%)	39 (54.2%)	0.0037 **
**Cause of sepsis**			
Pneumonia	41 (50.6%)	38 (52.8%)	0.7918
Peritonitis	21 (25.9%)	15 (20.8%)	0.4615
Gastrointestinal infection	4 (4.9%)	2 (2.7%)	0.4968
Wound infection	2 (2.5%)	3 (4.2%)	0.5608
Meningitis	1 (1.2%)	4 (5.5%)	0.1363
Endocarditis	1 (1.2%)	1 (1.4%)	0.9408
Other	11 (13.6%)	12 (16.7%)	0.5972
**Septic organ failure**			

**Multi-organ failure (≥2 organs)**	**41(50.7%)**	**37 (51.2%)**	**0.9260**
**Single organ failure**	**40 (49.3%)**	**35 (48.8%)**	**0.9260**
**RF**	**14 (17.3%)**	**14 (19.4%)**	**0.7330**
**ARDS**	**5 (6.24%)**	**4 (5.6%)**	**0.8753**
**ARF**	**7 (8.6%)**	**3 (4.2%)**	**0.2670**
**Other**	**14 (17.3%)**	**12 (16.7%)**	**0.9217**
**Disease Severity Scoring**			
**APACHE II**	**25.1 ± 8.2**	**30.37 ± 8.8**	**0.0002** ***
**SOFA**	**9.6 ± 4.8**	**11.4 ± 5.5**	**0.0151** *
**GCS**	**8.2 ± 3.2**	**7.9 ± 3.1**	**0.6672**
**Outcome**			
**Survivor (28 d)**	**50 (61.7%)**	**21 (37.5%)**	**0.0029** **

Data presented by number (%) or means ± SD.

Abbreviations: RF, respiratory failure; ARDS, acute respiratory distress syndrome; ARF, acute renal failure; ALF, acute liver failure; AHF, acute heart failure; APACHE II, acute physiology and chronic health evaluation II; SOFA, sequential organ failure assessment; GCS, Glasgow coma scale

Supplementary Table 3. The analysis of the patient characteristics. The IMD levels lower than 171 pg/ml were associated with older age (p=0.0174), a greater possibility of being accompanied by shock

(p=0.0037), higher Acute Physiology and Chronic Health Evaluation II (APACHE II, p=0.0002) and Sequential Organ Failure Assessment (SOFA, p=0.0151) scores, and higher mortality (p=0.0029).
